# Chitinivorax: The New Kid on the Block of Bacterial 2-Alkyl-4(1*H*)-quinolone Producers

**DOI:** 10.1021/acschembio.5c00046

**Published:** 2025-03-27

**Authors:** Viktoriia Savchenko, Xiaoqian Annie Yu, Martin F. Polz, Thomas Böttcher

**Affiliations:** † Faculty of Chemistry, Institute for Biological Chemistry & Centre for Microbiology and Environmental Systems Science, Department of Microbiology and Ecosystems Science, University of Vienna Josef-Holaubek-Platz 2 (UZA II), 1090 Vienna, Austria; ‡ Vienna Doctoral School in Chemistry (DoSChem), University of Vienna, Währinger Str. 42, 1090 Vienna, Austria; § Centre for Microbiology and Environmental Systems Science, Division of Microbial Ecology, University of Vienna, 1030 Vienna, Austria

## Abstract

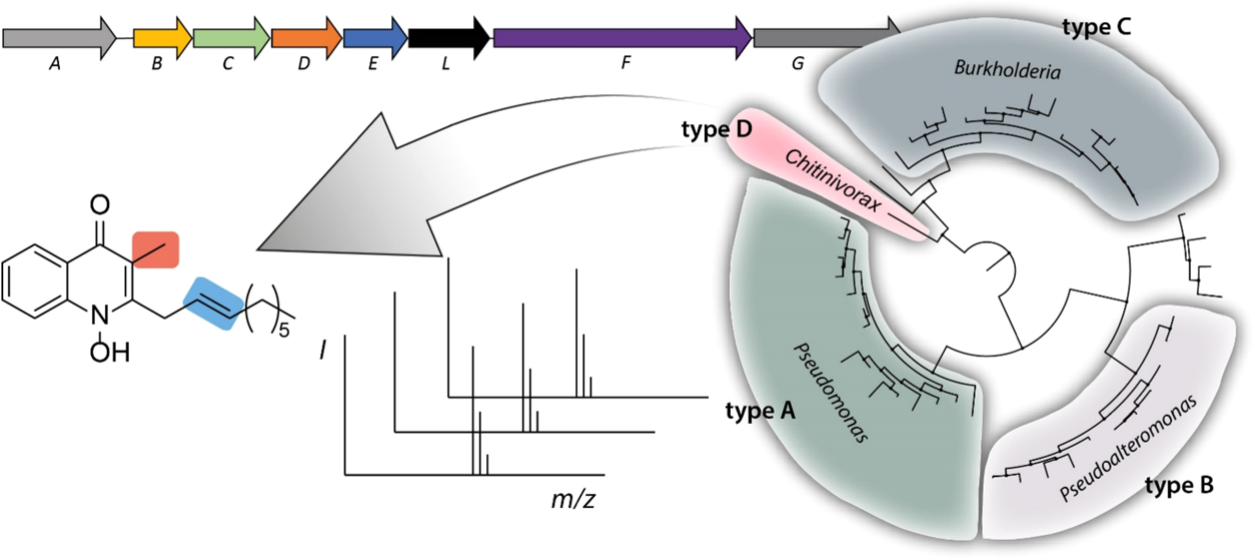

2-Alkyl-4(1*H*)-quinolones play a key role in bacterial communication, regulating biofilm formation, and virulence. Their antimicrobial properties also support bacterial survival and interspecies competition in microbial communities. In addition to the human pathogen *Pseudomonas aeruginosa* various species of *Burkholderia* and *Pseudoalteromonas* are known to produce 2-alkyl-4(1*H*)-quinolones. However, the evolutionary relationships of their biosynthetic gene clusters remain largely unexplored. To address this, we investigated the phylogeny of 2-alkyl-4(1*H*)-quinolone biosynthetic gene clusters, leading to the discovery of *Chitinivorax* as a fourth genus capable of producing 2-alkyl-4(1*H*)-quinolones, expanding our knowledge of the diversity of bacteria involved in quinolone-biosynthesis.

## Introduction

Quorum sensing (QS) is a mechanism through which bacteria regulate gene expression in dependence of population density, utilizing diffusible signaling molecules.^1,2^ QS regulates various physiological activities of microbial populations, including secondary metabolite production, biofilm formation, and secretion of virulence factors such as proteases and toxins.^3−5^

**Figure 1:**
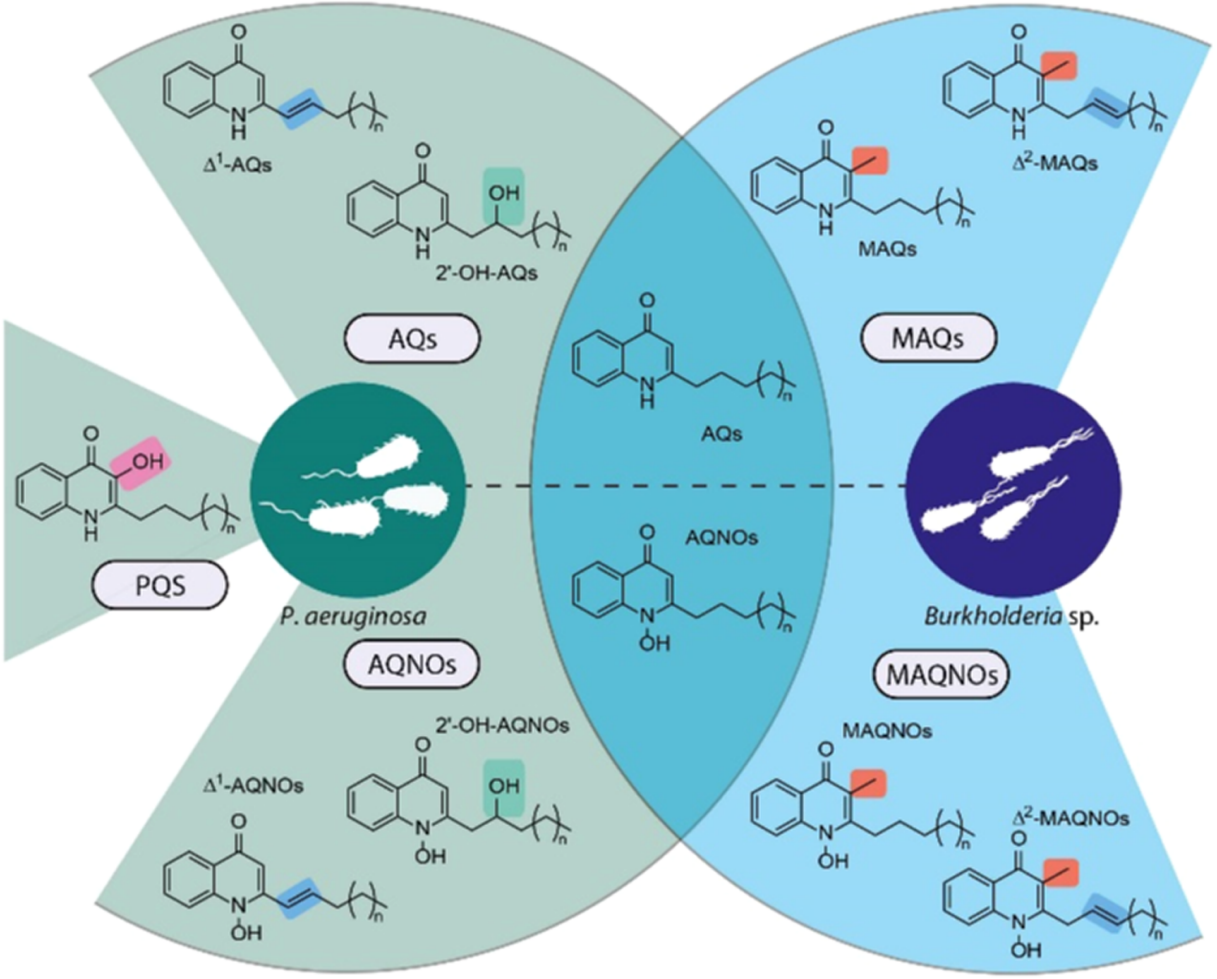
Various quinolone classes produced by *P. aeruginosa* and quinolone-producing *Burkholderia* spp.: Δ^1^-AQs (*trans*-Δ^1^-unsaturated 2-alkyl-4(1*H*)-quinolones), Δ^1^-AQNOs (*trans*-Δ^1^-unsaturated 2-alkyl-4(1*H*)-quinolone *N*-oxides), 2′–OH-AQs (2′-hydroxy-2-alkyl-4(1*H*)-quinolones), 2′–OH-AQNOs (2′-hydroxy-2-alkyl-4(1*H*)-quinolone *N*-oxides), AQs (2-alkyl-4(1*H*)-quinolones), AQNOs (2-alkyl-4(1*H*)-quinolone *N*-oxides), MAQs (3-methyl-2-alkyl-4(1*H*)-quinolones), MAQNOs (3-methyl-2-alkyl-4(1*H*)-quinolone *N*-oxides), Δ^2^-MAQs (*trans*-Δ^2^-unsaturated 3-methyl-2-alkyl-4(1*H*)-quinolones), Δ^2^-MAQNOs (*trans*-Δ^2^-unsaturated 3-methyl-2-alkyl-4(1*H*)-quinolone *N*-oxides).

Diverse microbial species employ QS, often encoding one or multiple classes of quorum sensing circuits. While some signals, such as acyl homoserine lactones (AHLs), are common across many proteobacteria, others are thought to be more exclusive to specific species. One notable example is the quinolone QS system of *Pseudomonas aeruginosa* using the Pseudomonas quinolone signal (PQS) and its biosynthetic precursor 2-heptyl-4(1*H*)-quinolone (HHQ). In total, *P. aeruginosa* produces over 50 different quinolones,^6^ mostly congeners of HHQ, i.e., 2-alkyl-4(1*H*)-quinolones (AQs), as well as 2-alkyl-4(1*H*)-quinolone *N*-oxides (AQNOs) varying in chain lengths and degrees of side chain saturation or hydroxylation (Figure 1).^7,8^ While AQNOs do not play a role in QS, they are potent antimicrobial weapons against competing microbial species like *Staphylococcus aureus* and are produced by the same biosynthetic gene cluster (BGC) as HHQ.^7,9^ Also HHQ and PQS exhibit diverse roles beyond signaling, ranging from trapping of ferric iron to immune modulatory and antimicrobial activities.^10−12^

Interestingly, members of the genus *Burkholderia* synthesize mainly methylated quinolone and quinolone *N*-oxide congeners, 3-methyl-2-alkyl-4(1*H*)-quinolones (MAQs) and 3-methyl-2-alkyl-4(1*H*)-quinolones *N*-oxides (MAQNOs), respectively (Figure 1).^7,13^ Unsaturated quinolones produced by *Burkholderia* have a different position of the unsaturation in side chains compared to AQs and AQNOs of *P. aeruginosa* (Figure 1). In addition, also *Pseudoalteromonas* species are capable of 2-alkyl-4(1*H*)-quinolone production and comprise a homologous BGC.

Remarkably, only a few species within the three genera *Pseudomonas*, *Pseudoalteromonas*, and *Burkholderia* were reported capable of producing AQ-type secondary metabolites.^6,14−16^ In this work, we aimed to examine the phylogeny of 2-alkyl-4(1*H*)-quinolone BGCs and discovered *Chitinivorax* as fourth genus producing 2-alkyl-4(1*H*)-quinolones.

## Results and Discussion

### Genome Mining for 2-Alkyl-4(1*H*)-quinolone Producers

**Figure 2:**
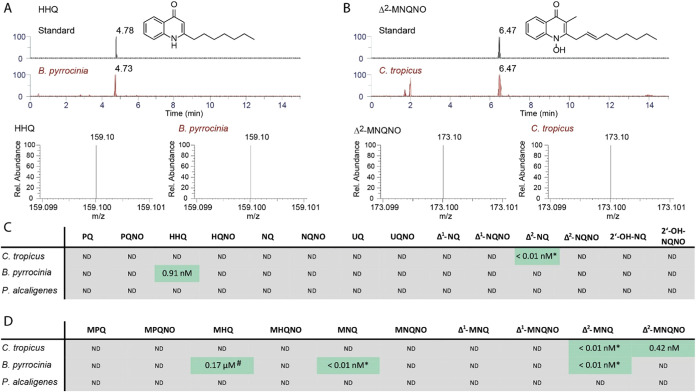
Detection of 2-alkyl-4(1*H*)-quinolone derivatives in culture supernatants of *Pseudoalteromonas alcaligenes*, *B. pyrrocinia*, and *C. tropicus*. (a) Identification of quinolones by LC-MS/MS using synthetic standards via retention time and mass spectrum, exemplary for (a) HHQ in *B. pyrrocinia* and (b) Δ^2^-MNQNO in *C. tropicus*. (c) Quantification of different AQs and AQNOs and (d) of MAQs and MAQNOs. *P. alcaligenes* and *B. pyrrocinia*, were grown in LB medium. *C. tropicus* was grown in R2A medium. ND = not detected. ^#^Quantified based on the calibration curve of the structurally closest related quinolone standard (MNQ) *below the lower limit of quantification (LLOQ).

We started with the analysis of BGCs responsible for the production of bacterial 2-alkyl-4(1*H*)-quinolones in the three known genera of producers: *Pseudomonas*, *Pseudoalteromonas*, and *Burkholderia* (Figure 2). In order to discover new quinolone-producing species, we used AntiSmash ClusterBlast to detect bacterial species carrying homologous BGCs. We found three potential hits which we manually analyzed to find the position of relevant biosynthetic genes in their respective full genomes with the NCBI BLAST tool. Among these, there were two species not previously reported to produce quinolones, *Burkholderia pyrrocinia* and *Pseudomonas alcaligenes*. Unexpectedly, the third candidate belonged to an entirely different genus: *Chitinivorax tropicus*. This recently discovered chitinolytic bacterium belongs to betaproteobacteria, is not yet assigned to a family, and originates from a freshwater lake in Taiwan.^17^

### LC-MS/MS Analysis of Quinolone Production

We obtained representative isolates of all three species from the German Collection of Microorganisms and Cell Cultures (DSMZ) in order to investigate their potential to produce 2-alkyl-4(1*H*)-quinolones. In earlier studies, our group could compare quinolone concentrations in bacterial extracts of *P. aeruginosa* PAO1 and PA14, *Burkholderia thailandensis*, and *Burkholderia ambifaria*, by using consistent growth conditions and LC-MS/MS techniques.^7,8,18^ Both *P. aeruginosa* strains as well as *B. thailandensis* were found to produce quinolones at concentrations of up to a few milligrams per liter (or μM level), while *B. ambifaria* produced them at significantly lower μg L^–1^ (or nM) levels.

Various incubation times and temperatures were analyzed (SI) but none of the three bacterial species were found to produce quinolones under this protocol. Suspecting that quinolone production might be as low as in the case of *B. ambifaria*, we switched to 12 times concentrated samples which were subjected to LC-MS/MS analysis. As a result, we could detect that *C. tropicus* produces *trans*-Δ^2^-unsaturated 2-nonyl-4(1*H*)-quinolone (Δ^2^-NQ), *trans*-Δ^2^-unsaturated 3-methyl-2-nonyl-4(1*H*)-quinolone (Δ^2^-MNQ) along with its *N*-oxide (Δ^2^-MNQNO), with the latter being the most abundant (0.13 μg L^–1^ or 0.42 nM).

We also identified that *B. pyrrocinia* synthesizes 3-methyl-2-heptyl-4(1*H*)-quinolone (MHQ), *trans*-Δ^2^-unsaturated 3-methyl-2-heptyl-4(1*H*)-quinolone (Δ^2^-MHQ), 3-methyl-2-nonyl-4(1*H*)-quinolone (MNQ), and the most prevalent 2-heptyl-4(1*H*)-quinolone (HHQ) (0.22 μg L^–1^ or 0.91 nM), which is usually used by quinolone-producing *Burkholderia* spp. as a QS signal.^19^ However, under the identical growth conditions and sample preparation protocol, we were unable to detect any quinolones in culture supernatants of *P. alcaligenes*.

Although the production of 2-alkyl-4(1*H*)-quinolones could be demonstrated in only two of the three species, the discovery of *Chitinivorax* as a new quinolone-producing genus is of great importance as it indicates a broader environmental distribution of this important metabolite class.

### Analysis of Quinolone Biosynthesis Genes

**Figure 3:**
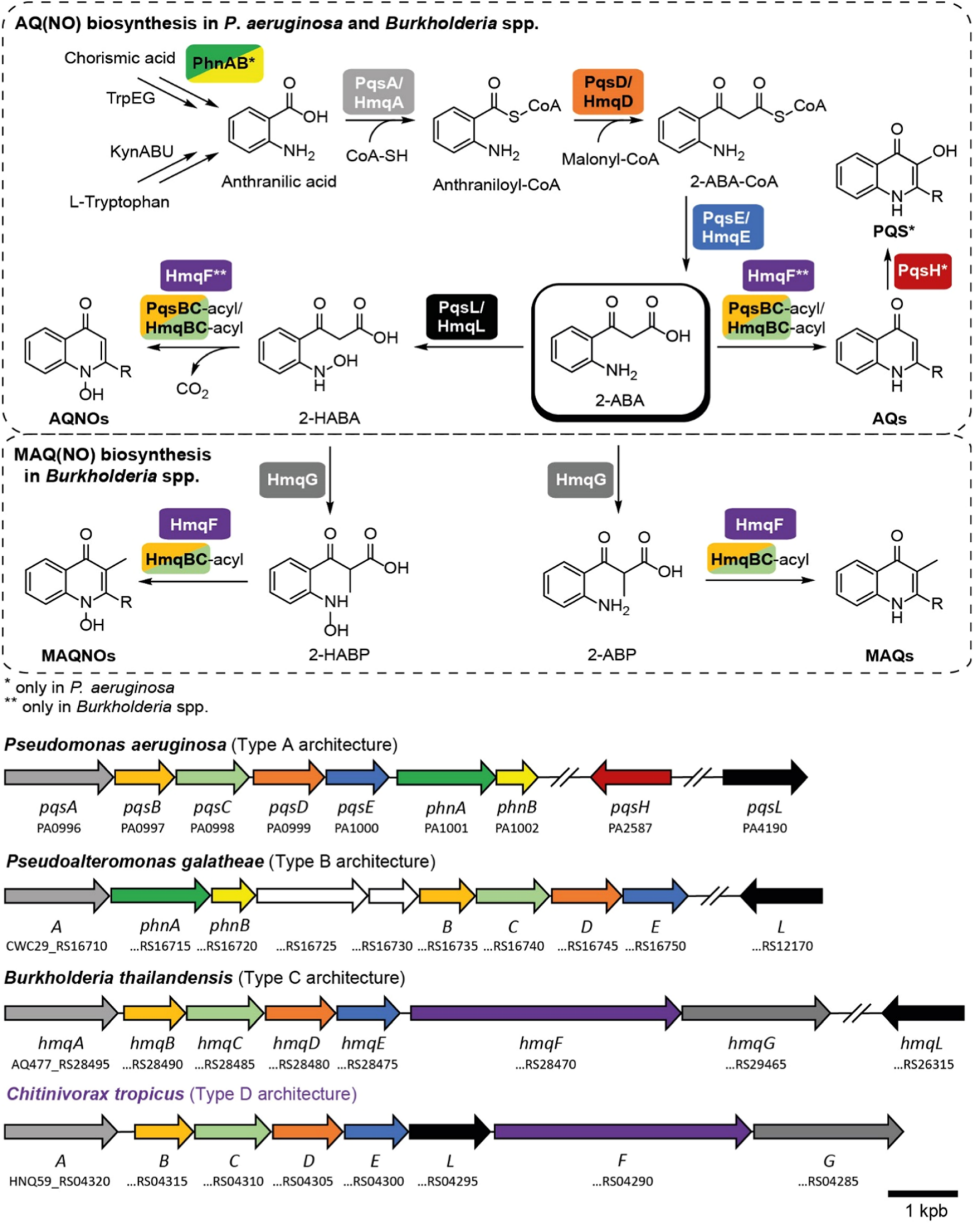
Scheme of the 2-alkyl-4(1*H*)-quinolone biosynthesis in *P. aeruginosa* and *B. thailandensis* with all key enzymes color coded according to the corresponding biosynthetic genes. Comparison of the four types of genetic architectures A–D with genes color coded corresponding to the enzymes for the biosynthesis of 2-alkyl-4(1*H*)-quinolones. *only for *P. aeruginosa*; **only for *Burkholderia* spp.

To investigate the biosynthetic basis for the production of quinolones of *C. tropicus*, we located and compared the genes of the respective BGC with that of other 2-alkyl-4(1*H*)-quinolone producers. As representative species of these genera, we selected *P. aeruginosa*, *Pseudoalteromonas galatheae*, and *B. thailandensis* and used the microbial genomes available at the National Center for Biotechnology Information (NCBI) database (SI). The biosynthetic pathways of 2-alkyl-4(1*H*)-quinolones have been investigated in detail for *Pseudomonas* and *Burkholderia* species and are shown in Figure 3a. In *P. aeruginosa*, the biosynthesis of AQs and AQNOs involves the *pqsABCDE* operon as well as the genes *pqsH* and *pqsL*. PqsA operates as an anthranilate-CoA ligase, activating anthranilate as anthraniloyl-CoA.^20,21^ Anthranilate can be generated by three different pathways: *phnAB*, *trpEG*, and kynurenine pathway.^20,22,23^ Notably, only *phnAB* transcription is directly under control of the PqsR regulator.^24,25^ In the second step of 4-quinolone biosynthesis, PqsD catalyzes the Claisen condensation of anthraniloyl-CoA and malonyl-CoA to 2-aminobenzoylacetate-CoA (2-ABA-CoA).^26,27^ Subsequently, PqsE acts as a thioesterase, performing the hydrolysis of 2-ABA-CoA into free 2-ABA. Optionally, PqsL, a FAD-dependent monooxygenase, oxidizes 2-ABA into 2-hydroxyaminobenzoylacetate (2-HABA).^28^

The heterodimeric PqsBC complex, ultimately catalyzes a Claisen condensation reaction between acyl-ACP and either 2-ABA or 2-HABA, resulting in the production of AQs or AQNOs, respectively.^28^ The *Pseudomonas*-unique FAD-dependent monooxygenase PqsH performs an additional oxidation of the C-3 position of HHQ leading to PQS.^29^ The *phnAB* operon, responsible for anthranilate synthesis, is located immediately after *pqsABCDE*. This represents the archetype of the quinolone biosynthetic machinery of *Pseudomonas* organized in a contiguous *pqsABCDE operon* followed by *phnAB* and the two single genes *pqsH* and *pqsL* that are located elsewhere in the genome. The *pqsH* gene is exclusive to *P*. *aeruginosa*, enabling it to produce the PQS signal. We classify this architecture as type A.

Remarkably, in *P. galatheae*, PqsA and PqsB encoding genes are not contiguous, unlike in *P. aeruginosa* and *B. thailandensis* (Figure 3a). A distinctive feature of quinolone-producing members of the *Pseudoalteromonas* genus is a gene region between *pqsA* and *pqsB* homologues, comprising four open reading frames (Figure 3a). For the identification of the most closely related homologues of these proteins in *P. aeruginosa*, we used protein BLAST analysis. CWC29_RS16715 and CWC29_RS16720 gene regions in *P. galatheae* were found to encode homologues of PhnA and PhnB, respectively. These resulted in 70 and 95% query coverage as well as 36.41 and 41.71% identity with *P. aeruginosa*’s PhnA and PhnB, respectively (Figure 3b). This suggests that anthranilate production genes are part of the quinolone BGC in *Pseudoalteromonas*, which is in agreement with results reported by Harvey et al. for multiple other *Pseudoalteromonas* species.^30^ The *phnAB* homologues were trailed by two genes with so far unknown function. A *pqsH* homologue is missing and a *pqsL* homologue is found as single gene at a separate location from the BGC in the genome. We classify this distinct topology of *Pseudoalteromonas* as type B architecture. Additionally, we could identify that *Pseudoalteromonas caenipelagi* and *Pseudoalteromonas aurantia* carry BGCs required for the quinolone biosynthesis, repeating typical for this genus type B architecture (SI). These species have neither been reported to carry quinolone biosynthesis-associated genes nor to produce typical AQs.

Burkholderia species use the *pqs* homologues *hmqABCDE* and two additional enzymes encoded by *hmqF* and *hmqG*.^31^ HmqF tailors the unsaturation of the side chain of MAQs and MAQNOs to the Δ^2^ position^32^ (Figure 1) and HmqG installs a methyl-group in 3-position of the quinolone core (Figure 2). We recently demonstrated that this methylation occurs at the stage of 2-ABA and 2-HABA.^31^ These genes are located right after *hmqE* resulting in the *hmqABCDEFG* operon. This architecture will be here classified as type C.

Notably, BLAST analysis revealed that *B. thailandensis* does not carry a homologue for *phnAB*, contrary to *Pseudoalteromonas* quinolone-producing species. It was reported earlier that *Burkholderia pseudomallei* also has no *phnAB* homologue and utilizes the *trpEG* and kynurenine pathways to generate anthranilate.^33^ During our analysis, we could determine for the first time that *B. pyrrocinia* (part of *Burkholderia cepacia* complex) as well as the newly discovered *Burkholderia mayonis* (part of *B. pseudomallei* complex) both contain quinolone biosynthesis-associated genes, in type C architecture (Figure S1b).

*C. tropicus* comprises an architecture that topologically resembles type C of *Burkholderia* and even contains *hmqF* and *hmqG* homologues, which were previously considered unique to quinolone-producing *Burkholderia*. Similar to *Burkholderia*, we also could not find *phnA* and *phnB* homologues in *C. tropicus*. Contrary to *Burkholderia* spp., *C. tropicus* has a more noticeable gap between *hmqA* and *hmqB* homologues. However, the most distinctive feature compared to the type C architecture is the unique position of the *hmqL*/*pqsL* homologue (Figure 3a). *Chitinivorax* is the only example, where the *hmqL/pqsL* homologue responsible for the production of quinolone *N*-oxides is an actual part of the quinolone BGC. In *C. tropicus*, the *hmqL/pqsL* homologue is found between *hmqE* and *hmqF* homologues, locating all quinolone-responsible genes in one cluster with a new unique *ABCDE***L***FG* architecture, which we classify here as type D (Figure 3b). The unusual position of the *pqsL*/*hmqL* homologue within the BGC may explain the observation that predominantly the *N*-oxide Δ^2^-MNQNO was produced (0.42 nM) while all other quinolones detected were produced at least at 40-fold lower concentrations below the lower limit of quantification (<0.01 nM) (Figure 2c,d). The occurrence of an N-oxide as the major metabolite is unusual and typically (M)AQs are produced at comparable levels as (M)AQNOs in *Pseudomonas* and *Burkholderia*.^7^ It may be thus hypothesized that having the corresponding *N*-oxidation enzyme as part of the BGC streamlines the production toward quinolone *N*-oxides.

The four genetic architectures were found to be highly consistent with the four genera and we did not discover any species of a genus with a cross-type architecture.

Having defined the four different biosynthetic architectures, we next aimed to investigate their phylogeny and potential evolutionary trajectory.

### Phylogeny of Quinolone Biosynthesis Genes

**Figure 4:**
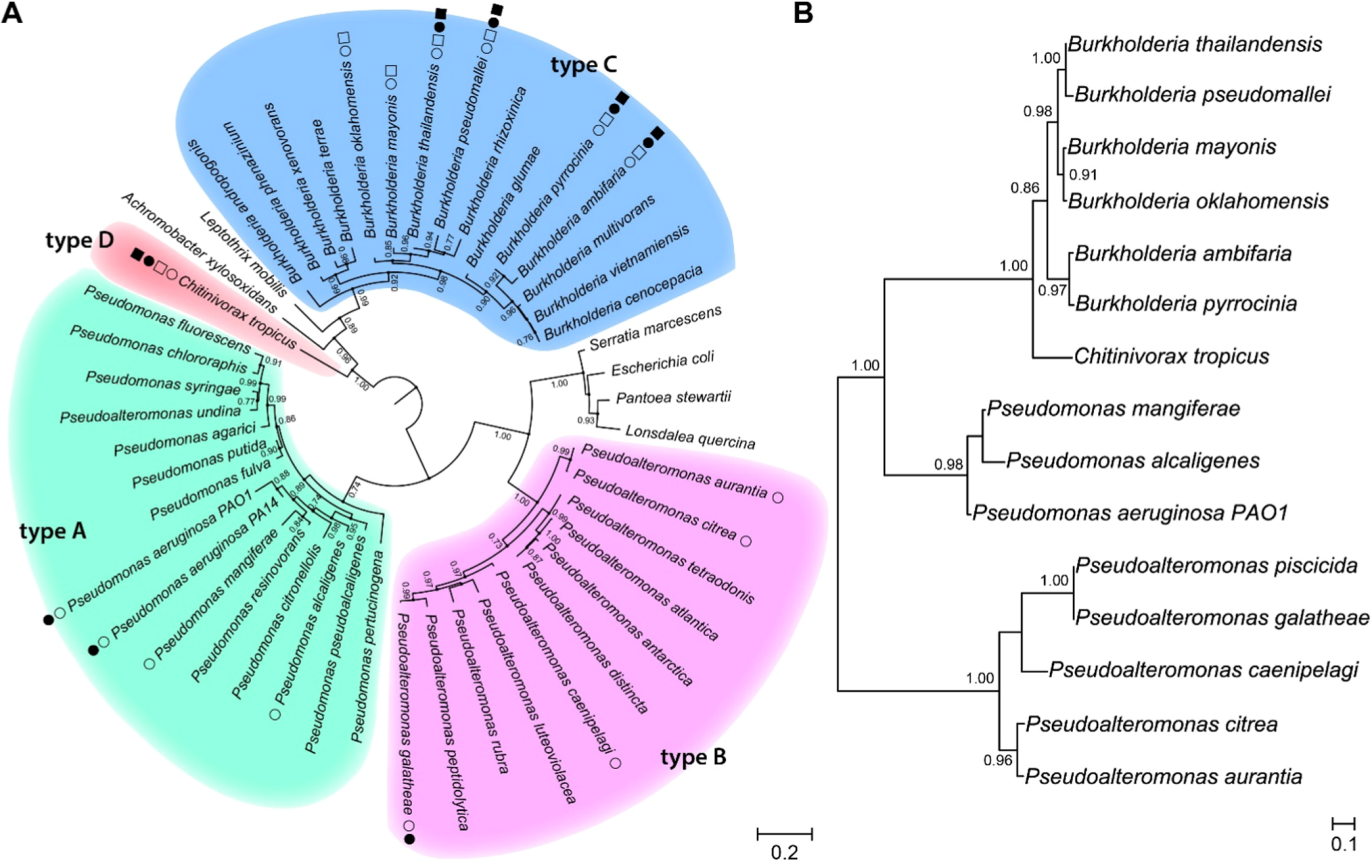
(A) Distribution of biosynthetic capabilities for 2-alkyl-4(1*H*)-quinolones is mapped onto a 16S rRNA gene phylogenetic tree. Genomes from each taxonomic unit were analyzed for the presence of 2-alkyl-4(1*H*)-quinolone BGCs, represented by empty circles. Empty squares denote the presence of *hmqFG* homologues. Filled circles indicate experimentally confirmed AQ or AQNO production, while filled squares denote MAQ or MAQNO production. Architectures types are denoted by A–C. The evolutionary history was inferred using the maximum likelihood method.^37^ Branch support values, indicated at the nodes, were calculated using the Shimodaira–Hasegawa approximate likelihood ratio test (SH-aLRT).^38^ To enhance readability, only support values greater than 0.7 are displayed on the tree. (B) Protein tree of pqsD homologues show congruent phylogeny with the 16S rDNA tree. Both trees in (A, B) are midpoint rooted.

To examine the phylogenetic position of *Chitinivorax* in in the relation to other 2-alkyl-4(1*H*)-quinolone producers, we built a 16S rDNA phylogenetic maximum likelihood tree with PhyML (Figure 4A) and marked the presence of quinolone BGCs as well as the confirmed production of the corresponding secondary metabolites. The distribution of the BGCs largely does not follow a phylogenetic pattern within and between clades suggesting that horizontal gene transfer was a major driving force for the dissemination of the biosynthetic capabilities for quinolone production. *Chitinivorax* is phylogenetically closer to the clade of *Burkholderia*, which is in agreement with the more similar architectures of types C and D of quinolone biosynthesis genes. However, *Chitinivorax* and *Burkholderia* form two distinct clades separated by other genera like *Achromobacter* and *Leptothrix* that do not encode any homologous BGC for quinolone production. These results indicate that the genes for 2-alkyl-4(1*H*)-quinolone biosynthesis are more widely distributed within the phylogenetic tree than previously known.

In order to investigate their evolutionary trajectory, we generated protein trees for individual enzymes involved in quinolone biosynthesis (Figures S2–S4). Homologues of Pqs/HmqBCDE display highly congruent phylogenies, indicating they have likely have traveled together (Figures 4b and S2). Interestingly, the PqsA homologue in *Chitinovorax* appears to have diverged from the rest of the cluster, likely originating through horizontal gene transfer from within the *Burkholderia* clade (Figure S2). Meanwhile, HmqFG are congruent with each other and with the formation of the *Chitinovorax*/*Burkholderia* clade on the 16S rDNA tree, suggesting a single acquisition event in the common ancestor of these two taxa, followed by vertical transmission (Figure S3). In contrast, HmqL/PqsL homologues show phylogenetic incongruence with the 16S rDNA tree, suggesting they may have experienced more complex trajectories of horizontal transfer and exchange (Figure S4).

Our study exclusively focuses on the biosynthesis of 2-alkyl-4(1*H*)-quinolones and does not include aurachin-like quinolones from *Streptomyces*, *Stigmatella* and *Rhodococcus*.^34−36^ Unlike 2-alkyl-4(1*H*)-quinolones, aurachins are synthesized through a distinct pathway involving the condensation of anthraniloyl-CoA with two malonyl-CoA molecules to form 2-methyl-4-hydroxyquinoline, which is subsequently modified by isoprenyl pyrophosphate.^36^ Consequently, aurachin biosynthesis relies on a significantly different set of enzymatic reactions.

## Conclusions

We have shown that 2-alkyl-4(1*H*)-quinolones, previously thought to be restricted to *Pseudomonas*, *Pseudoalteromonas*, and *Burkholderia*, are also produced by the genus *Chitinivorax*. These four genera feature considerably different architectures of the biosynthetic gene cluster and accessory genes and likely have been acquired and exchanged via horizontal gene transfer. 2-Alkyl-4(1*H*)-quinolones serve many important functions, including quorum sensing and antimicrobial defense. Our results suggest that quinolone production may be more widespread among different genera and species in the environment than previously thought.

## Supporting Information Available

Additional experimental details; materials, and methods; mass spectra and MS^2^ assignments and quantification of quinolones; additional details of phylogenetic analyses (PDF)

## References

[ref1] Fuqua W. C.; Winans S. C.; Greenberg E. P. Quorum sensing in bacteria: the LuxR-LuxI family of cell density-responsive transcriptional regulators. J. Bacteriol. 1994, 176, 269–275. 10.1128/jb.176.2.269-275.1994. 8288518 PMC205046

[ref2] Aframian N.; Eldar A. A Bacterial Tower of Babel: Quorum-Sensing Signaling Diversity and Its Evolution. Annu. Rev. Microbiol. 2020, 74, 587–606. 10.1146/annurev-micro-012220-063740. 32680450 PMC7611908

[ref3] Van Delden C.; Iglewski B. H. Cell-to-cell signaling and *Pseudomonas aeruginosa* infections. Emerging Infect. Dis. 1998, 4, 551–560. 10.3201/eid0404.980405. PMC2640238 9866731

[ref4] Calfee M. W.; Coleman J. P.; Pesci E. C. Interference with Pseudomonas quinolone signal synthesis inhibits virulence factor expression by Pseudomonas aeruginosa. Proc. Natl. Acad. Sci. U.S.A. 2001, 98, 11633–11637. 10.1073/pnas.201328498. 11573001 PMC58781

[ref5] Bjarnsholt T.; Tolker-Nielsen T.; Hoiby N.; Givskov M. Interference of Pseudomonas aeruginosa signalling and biofilm formation for infection control. Expert Rev. Mol. Med. 2010, 12, e11 10.1017/s1462399410001420. 20370936

[ref6] Lepine F.; Milot S.; Deziel E.; He J.; Rahme L. G. Electrospray/mass spectrometric identification and analysis of 4-hydroxy-2-alkylquinolines (HAQs) produced by Pseudomonas aeruginosa. J. Am. Soc. Mass Spectrom. 2004, 15, 862–869. 10.1016/j.jasms.2004.02.012. 15144975

[ref7] Szamosvári D.; Prothiwa M.; Dieterich C. L.; Böttcher T. Profiling structural diversity and activity of 2-alkyl-4(1H)-quinolone N-oxides of Pseudomonas and Burkholderia. Chem. Commun. 2020, 56, 6328–6331. 10.1039/D0CC02498H. 32436549

[ref8] Savchenko V.; Szamosvari D.; Bao Y.; Pignitter M.; Böttcher T. Biosynthetic flexibility of Pseudomonas aeruginosa leads to hydroxylated 2-alkylquinolones with proinflammatory host response. Commun. Chem. 2023, 6, 138 10.1038/s42004-023-00937-y. 37400564 PMC10318067

[ref9] Szamosvári D.; Böttcher T. An Unsaturated Quinolone N-Oxide of Pseudomonas aeruginosa Modulates Growth and Virulence of Staphylococcus aureus. Angew. Chem., Int. Ed. 2017, 56, 7271–7275. 10.1002/anie.201702944. 28523838

[ref10] Diggle S. P.; Matthijs S.; Wright V. J.; Fletcher M. P.; Chhabra S. R.; Lamont I. L.; Kong X.; Hider R. C.; Cornelis P.; Camara M.; Williams P. The Pseudomonas aeruginosa 4-quinolone signal molecules HHQ and PQS play multifunctional roles in quorum sensing and iron entrapment. Chem. Biol. 2007, 14, 87–96. 10.1016/j.chembiol.2006.11.014. 17254955

[ref11] Bredenbruch F.; Geffers R.; Nimtz M.; Buer J.; Haussler S. The Pseudomonas aeruginosa quinolone signal (PQS) has an iron-chelating activity. Environ. Microbiol. 2006, 8, 1318–1329. 10.1111/j.1462-2920.2006.01025.x. 16872396

[ref12] Lin J.; Cheng J.; Wang Y.; Shen X. The Pseudomonas Quinolone Signal (PQS): Not Just for Quorum Sensing Anymore. Front. Cell Infect. Microbiol. 2018, 8, 230 10.3389/fcimb.2018.00230. 30023354 PMC6039570

[ref13] Coulon P. M. L.; Groleau M. C.; Deziel E. Potential of the Burkholderia cepacia Complex to Produce 4-Hydroxy-3-Methyl-2-Alkyquinolines. Front. Cell. Infect. Microbiol. 2019, 9, 33 10.3389/fcimb.2019.00033. 30873388 PMC6403149

[ref14] Pesci E. C.; Milbank J. B.; Pearson J. P.; McKnight S.; Kende A. S.; Greenberg E. P.; Iglewski B. H. Quinolone signaling in the cell-to-cell communication system of Pseudomonas aeruginosa. Proc. Natl. Acad. Sci. U.S.A. 1999, 96, 11229–11234. 10.1073/pnas.96.20.11229. 10500159 PMC18016

[ref15] Kim W. J.; Kim Y. O.; Kim J. H.; Nam B. H.; Kim D. G.; An C. M.; Lee J. S.; Kim P. S.; Lee H. M.; Oh J. S.; Lee J. S. Liquid Chromatography-Mass Spectrometry-Based Rapid Secondary-Metabolite Profiling of Marine Pseudoalteromonas sp. M2. Mar. Drugs 2016, 14, 24 10.3390/md14010024. 26805856 PMC4728520

[ref16] Diggle S. P.; Lumjiaktase P.; Dipilato F.; Winzer K.; Kunakorn M.; Barrett D. A.; Chhabra S. R.; Camara M.; Williams P. Functional genetic analysis reveals a 2-Alkyl-4-quinolone signaling system in the human pathogen Burkholderia pseudomallei and related bacteria. Chem. Biol. 2006, 13, 701–710. 10.1016/j.chembiol.2006.05.006. 16873018

[ref17] Chen W. M.; Yang S. H.; Huang W. C.; Cheng C. Y.; Sheu S. Y. Chitinivorax tropicus gen. nov., sp. nov., a chitinolytic bacterium isolated from a freshwater lake. Int. J. Syst. Evol. Microbiol. 2012, 62, 1086–1091. 10.1099/ijs.0.031310-0. 21705448

[ref18] Prothiwa M.; Filz V.; Oehler S.; Böttcher T. Inhibiting quinolone biosynthesis of Burkholderia. Chem. Sci. 2021, 12, 6908–6912. 10.1039/D0SC06167K. 34123319 PMC8153077

[ref19] Vial L.; Lepine F.; Milot S.; Groleau M. C.; Dekimpe V.; Woods D. E.; Deziel E. *Burkholderia pseudomallei*, *B. thailandensis*, and *B. ambifaria* produce 4-hydroxy-2-alkylquinoline analogues with a methyl group at the 3 position that is required for quorum-sensing regulation. J. Bacteriol. 2008, 190, 5339–5352. 10.1128/JB.00400-08. 18539738 PMC2493281

[ref20] Gallagher L. A.; McKnight S. L.; Kuznetsova M. S.; Pesci E. C.; Manoil C. Functions required for extracellular quinolone signaling by Pseudomonas aeruginosa. J. Bacteriol. 2002, 184, 6472–6480. 10.1128/JB.184.23.6472-6480.2002. 12426334 PMC135424

[ref21] Coleman J. P.; Hudson L. L.; McKnight S. L.; Farrow J. M.; 3rd; Calfee M. W.; Lindsey C. A.; Pesci E. C. Pseudomonas aeruginosa PqsA is an anthranilate-coenzyme A ligase. J. Bacteriol. 2008, 190, 1247–1255. 10.1128/JB.01140-07. 18083812 PMC2238192

[ref22] Farrow J. M. 3rd; Pesci E. C. Two distinct pathways supply anthranilate as a precursor of the Pseudomonas quinolone signal. J. Bacteriol. 2007, 189, 3425–3433. 10.1128/JB.00209-07. 17337571 PMC1855905

[ref23] Kurnasov O.; Goral V.; Colabroy K.; Gerdes S.; Anantha S.; Osterman A.; Begley T. P. NAD biosynthesis: identification of the tryptophan to quinolinate pathway in bacteria. Chem. Biol. 2003, 10, 1195–1204. 10.1016/j.chembiol.2003.11.011. 14700627

[ref24] Cao H.; Krishnan G.; Goumnerov B.; Tsongalis J.; Tompkins R.; Rahme L. G. A quorum sensing-associated virulence gene of Pseudomonas aeruginosa encodes a LysR-like transcription regulator with a unique self-regulatory mechanism. Proc. Natl. Acad. Sci. U.S.A. 2001, 98, 14613–14618. 10.1073/pnas.251465298. 11724939 PMC64730

[ref25] Wade D. S.; Calfee M. W.; Rocha E. R.; Ling E. A.; Engstrom E.; Coleman J. P.; Pesci E. C. Regulation of Pseudomonas quinolone signal synthesis in Pseudomonas aeruginosa. J. Bacteriol. 2005, 187, 4372–4380. 10.1128/JB.187.13.4372-4380.2005. 15968046 PMC1151766

[ref26] Zhang Y. M.; Frank M. W.; Zhu K.; Mayasundari A.; Rock C. O. PqsD is responsible for the synthesis of 2,4-dihydroxyquinoline, an extracellular metabolite produced by Pseudomonas aeruginosa. J. Biol. Chem. 2008, 283, 28788–28794. 10.1074/jbc.M804555200. 18728009 PMC2570881

[ref27] Bera A. K.; Atanasova V.; Robinson H.; Eisenstein E.; Coleman J. P.; Pesci E. C.; Parsons J. F. Structure of PqsD, a Pseudomonas quinolone signal biosynthetic enzyme, in complex with anthranilate. Biochemistry 2009, 48, 8644–8655. 10.1021/bi9009055. 19694421 PMC2775144

[ref28] Drees S. L.; Ernst S.; Belviso B. D.; Jagmann N.; Hennecke U.; Fetzner S. PqsL uses reduced flavin to produce 2-hydroxylaminobenzoylacetate, a preferred PqsBC substrate in alkyl quinolone biosynthesis in Pseudomonas aeruginosa. J. Biol. Chem. 2018, 293, 9345–9357. 10.1074/jbc.RA117.000789. 29669807 PMC6005433

[ref29] Schertzer J. W.; Brown S. A.; Whiteley M. Oxygen levels rapidly modulate Pseudomonas aeruginosa social behaviours via substrate limitation of PqsH. Mol. Microbiol. 2010, 77, 1527–1538. 10.1111/j.1365-2958.2010.07303.x. 20662781 PMC3098721

[ref30] Harvey E. L.; Deering R. W.; Rowley D. C.; El Gamal A.; Schorn M.; Moore B. S.; Johnson M. D.; Mincer T. J.; Whalen K. E. A Bacterial Quorum-Sensing Precursor Induces Mortality in the Marine Coccolithophore, Emiliania huxleyi. Front. Microbiol. 2016, 7, 59 10.3389/fmicb.2016.00059. 26870019 PMC4737879

[ref31] Savchenko V.; Jaegers M.; Rasche R.; Böttcher T.; Fetzner S.; Ernst S.; et al. Unraveling key steps in the biosynthesis of antimicrobial methylated unsaturated 2-alkyl-4-quinolones of Burkholderia thailandensis. Cell Rep. Phys. Sci. 2024, 5, 102100 10.1016/j.xcrp.2024.102100.

[ref32] Agarwal A.; Kahyaoglu C.; Hansen D. B. Characterization of HmqF, a protein involved in the biosynthesis of unsaturated quinolones produced by Burkholderia thailandensis. Biochemistry 2012, 51, 1648–1657. 10.1021/bi201625w. 22320268

[ref33] Butt A.; Halliday N.; Williams P.; Atkins H. S.; Bancroft G. J.; Titball R. W. Burkholderia pseudomallei kynB plays a role in AQ production, biofilm formation, bacterial swarming and persistence. Res. Microbiol. 2016, 167, 159–167. 10.1016/j.resmic.2015.11.002. 26654915

[ref34] Pistorius D.; Li Y.; Sandmann A.; Müller R. Completing the puzzle of aurachin biosynthesis in Stigmatella aurantiaca Sg a15. Mol. Biosyst. 2011, 7, 3308–3315. 10.1039/c1mb05328k. 21979787

[ref35] Kitagawa W.; Tamura T. A quinoline antibiotic from Rhodococcus erythropolis JCM 6824. J. Antibiot. 2008, 61, 680–682. 10.1038/ja.2008.96. 19168983

[ref36] Zhang M.; Yang C. L.; Xiao Y. S.; Zhang B.; Deng X. Z.; Yang L.; Shi J.; Wang Y. S.; Li W.; Jiao R. H.; Tan R. X.; Ge H. M. Aurachin SS, a new antibiotic from Streptomyces sp. NA04227. J. Antibiot. 2017, 70, 853–855. 10.1038/ja.2017.50. 28420868

[ref37] Guindon S.; Dufayard J. F.; Lefort V.; Anisimova M.; Hordijk W.; Gascuel O. New algorithms and methods to estimate maximum-likelihood phylogenies: assessing the performance of PhyML 3.0. Syst. Biol. 2010, 59, 307–321. 10.1093/sysbio/syq010. 20525638

[ref38] Anisimova M.; Gascuel O. Approximate likelihood-ratio test for branches: A fast, accurate, and powerful alternative. Syst. Biol. 2006, 55, 539–552. 10.1080/10635150600755453. 16785212

