# Supplementary material for: Chitinivorax: The New Kid on the Block of Bacterial 2-Alkyl-4(1H)-quinolone Producers
Source: ACS Chem Biol. 2025 Mar 27;20(4):960–6. doi: 10.1021/acschembio.5c00046 (PMC12012761; doi:10.1021/acschembio.5c00046)
Supplement: Supplementary file 1 — cb5c00046_si_001.pdf [file cb5c00046_si_001.pdf]

Supporting Information

Chitinivorax: The New Kid on the Block of Bacterial 2-Alkyl-4(1H)-quinolone Producers

Viktoriia Savchenko,[a,b] Xiaoqian Annie Yu[c], Martin Polz[c] and Thomas Böttcher*[a,d]

Faculty of Chemistry, Institute for Biological Chemistry & Centre for Microbiology and Environmental Systems Science, Department of Microbiology and Ecosystems Science, University of Vienna
Josef-Holaubek-Platz 2 (UZA II), 1090 Vienna, Austria

Vienna Doctoral School in Chemistry (DoSChem), University of Vienna, Währinger Str. 42, 1090 Vienna, Austria

Centre for Microbiology and Environmental Systems Science, Division of Microbial Ecology, University of Vienna

Environment and Climate Research Hub (ECH), University of Vienna, Augasse 2-6, 1090 Vienna. Austria
email: thomas.boettcher@univie.ac.at

Table of Contents

1. Methods 2
2. MS^2 assignments 4
3. Tables 12
4. Figures 20
5. References 30

1. Methods

Preparation of overnight cultures

A small amount of a bacterial cryo-stock (20% glycerol, stored at -80 °C) was inoculated in 3 mL LB (lysogeny broth) (LB medium) in sterile 13 mL polypropylene tubes (Sarstedt, ref 62.515.028), and allowed to grow for 16-18 h at indicated growth conditions (**Table S1**).

Quantification of quinolones in bacterial cultures

An overnight culture (450 µL) was inoculated into 30 mL of LB medium in sterile 50 mL polypropylene centrifugal tubes with screw caps (VWR). Caps of the tubes were loosely opened by a 180-degree turn and fixed in this position to ensure equal oxygen supply. Cultures were incubated for 16-24 h at 37˚C in a shaking incubator at 180 rpm. After incubation, samples were centrifuged at 4500 rpm for 10 min and supernatants were sterile filtrated. 6 mL of culture supernatant was added in 10 mL glass vials, followed by 4 mL of EtOAc and the mixture was immediately vortexed for 5 sec. After the separation of organic and water phases, 250 µL of the EtOAc layer was transferred via pipetting into mass spec vials. The EtOAc was evaporated by a gentle stream of nitrogen. This procedure was repeated 8 times to concentrate 2 mL of extract (8x250 µL). For LC-MS/MS analysis, 250 µL of sample solvent (MeOH/H_2O 1:1) was added to glass vials and the residue was redissolved. Then 100 µL of this solution was transferred via pipetting into mass spec vials containing a glass insert (MACHEREY-NAGEL, Art. Nr. 702007) and subjected to LC-MS/MS analysis. The experiment was performed in triplicates.

Calibration curves for quantification

MeOH stocks of all calibration standards were prepared at 1 mg/mL concentration in glass vials and stored at -80°C for up to 3 months. Calibration standard samples for quantification were prepared in triplicates by serial dilution in sample solvent (MeOH/H_2O 1:1) (**Table S2**). Calibration equations and R-values were obtained using the Thermo Xcalibur Quan Browser.

LC-MS/MS analysis

LC-MS/MS analysis was performed as described by Prothiwa et al.[1]

Quinolone standards were synthesized as described by Szamosvari et al.[2]
Ultra-high performance liquid chromatography was performed on a Vanquish™ UHPLC system (Thermo Fisher Scientific) using a Nucleodur C18 Gravity-SB 100 x 2 mm, 3 µm column (Macherey-Nagel). The flow rate was 0.5 mL min^{-1} and the column temperature was held at 40°C. The injection volume was 10 µL. Eluent A was 0.1% formic acid in water and eluent B was 0.1% formic acid in acetonitrile. The gradient was 20-100 % B in 10 min, 100 % B for 2 min, 100-20 % B in 1 min, and 20 % B for 2 min. MS/MS analysis was performed by TSQ® Series II Quantum (Thermo Fisher Scientific) mass spectrometer. A heated-electrospray ionization (HESI-II probe, Thermo Scientific) was used as an ion source. In the optimized conditions the ion spray voltage was 3500 V, vaporizer temperature 300°C, capillary temperature 380°C, sheath gas pressure 60 psi, ion sweep gas pressure 2 psi, and aux gas 10 psi. The fragmentation pattern of quinolone standards was acquired in a product ion scan mode using a fixed collision energy of 30 V to fragment the corresponding precursor ion before recording the fragments in a mass range of m/z 130-350. Quinolones we quantified in Selected Reaction Monitoring (SRM) scan mode. MS/MS spectra were acquired in a positive mode. The software Quan Browser Thermo Xcalibur was used for quantitative analysis. The peak area of the respective product ion was fitted by linear regression versus the known concentrations to generate a standard curve.

Phylogenetic analysis
The 16s rDNA sequences of 48 bacterial genomes were aligned with MUSCLE.[3] The alignment was used to construct a maximum-likelihood tree using PhyML (v3.3)[4] with a General Time Reversible substitution model, as well as empirical models for the equilibrium nucleotide frequency, nucleotide substitution rates, and the gamma distribution shape parameter. Branch support values were calculated using the Shimodaira-Hasegawa approximate likelihood ratio test (SH-aLRT).[5]

Protein sequence analysis
The protein sequences for PqsA, PqsB, PqsC, PqsD, or PqsE and their homologs were aligned respectively with MUSCLE. The evolutionary history was inferred by using the maximum-likelihood method and JTT matrix-based model[3] in MEGA[6]. The bootstrap consensus tree inferred from 1000 replicates[7] is taken to represent the evolutionary history of the taxa analyzed. Branches corresponding to partitions reproduced in less than 50% bootstrap replicates are collapsed. Initial tree(s) for the heuristic search were obtained automatically by applying Neighbor-Join and BioNJ algorithms to a matrix of pairwise distances estimated using the JTT model, and then selecting the topology with superior log likelihood value. A discrete Gamma distribution was used to model evolutionary rate differences among sites (5 categories (+*G*, parameter = 1.3721)). The rate variation model allowed for some sites to be evolutionarily invariable ([+*I*], 15.35% sites). All positions containing gaps and missing data were eliminated (complete deletion option).

2. MS^2 assignments

Reference spectra (Product Ion Scan)

HHQ (RT = 4.71)

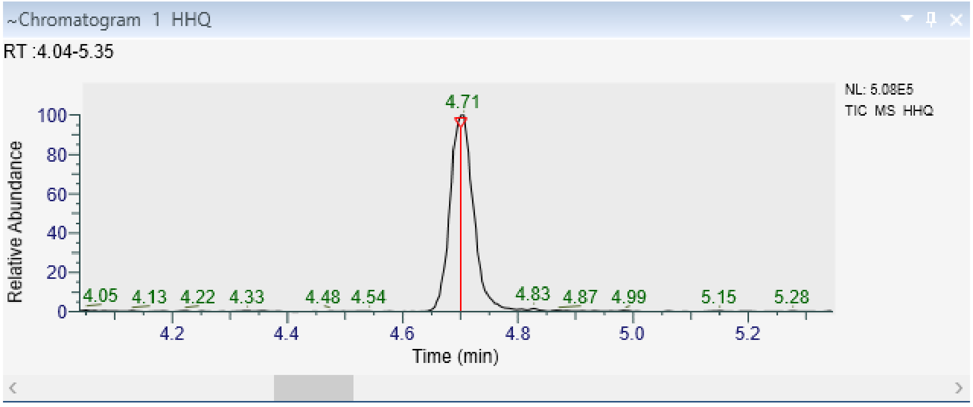

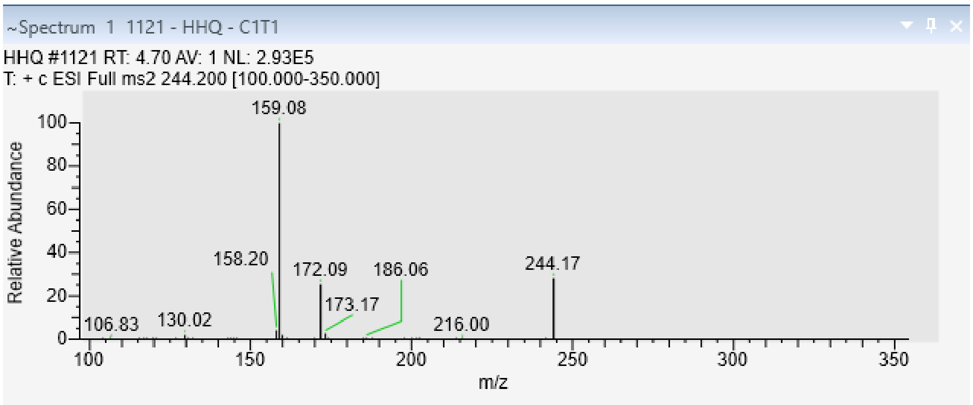

Δ^2-NQ (RT = 5.79)

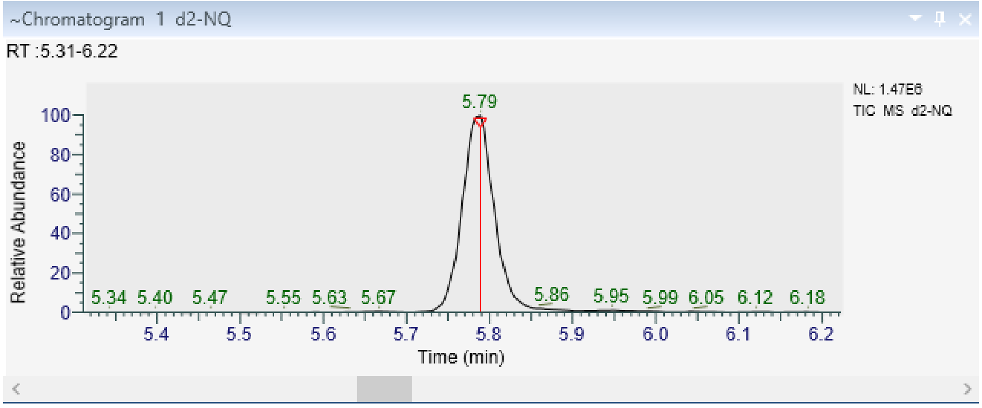

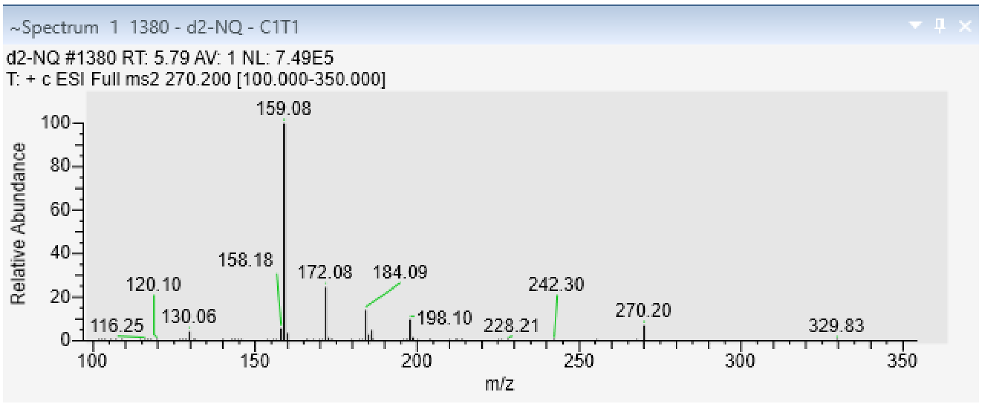

MNQ (RT = 6.88)

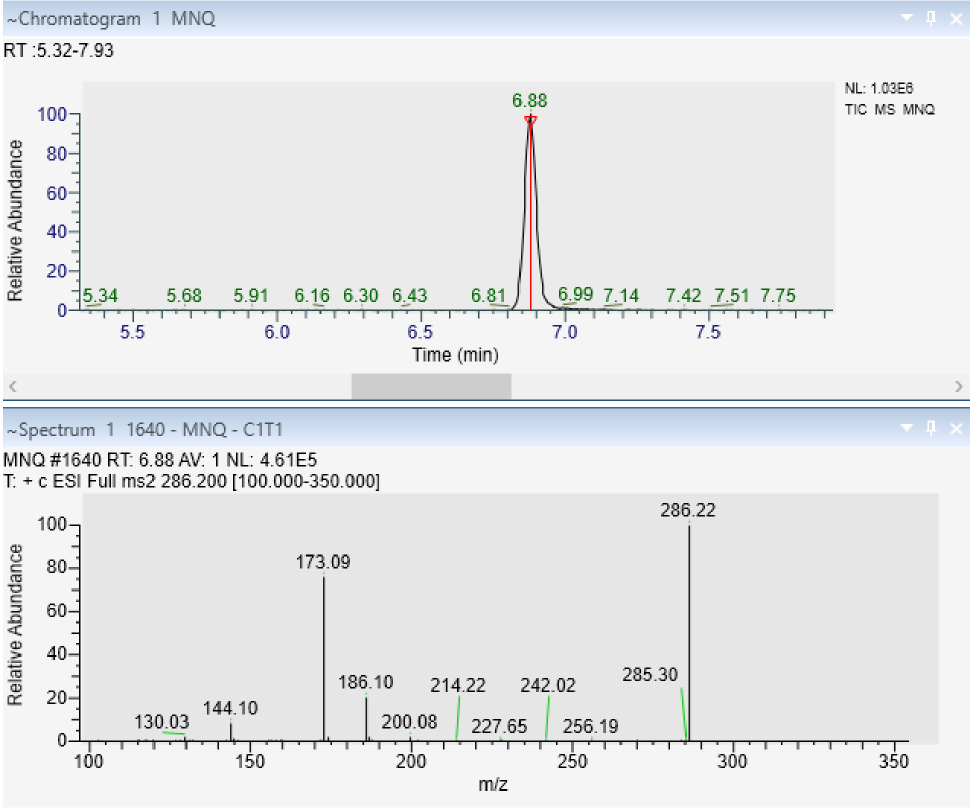

Δ^2-MNQ (RT = 6.52)

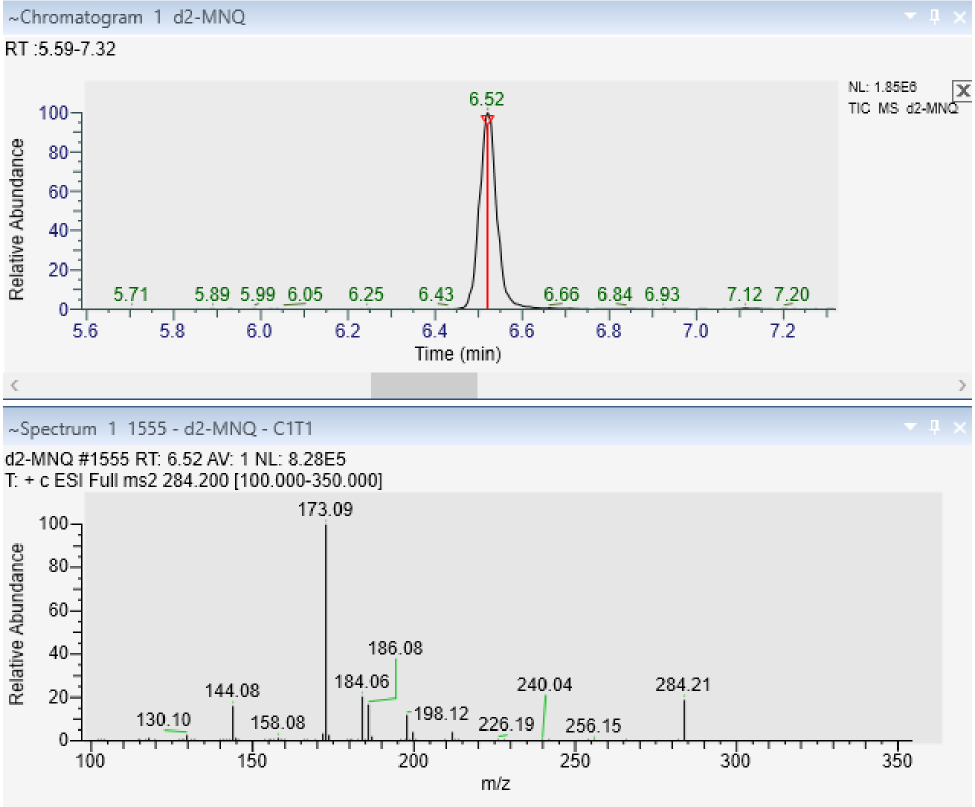

Δ[2]-MNQNO (RT = 6.47)

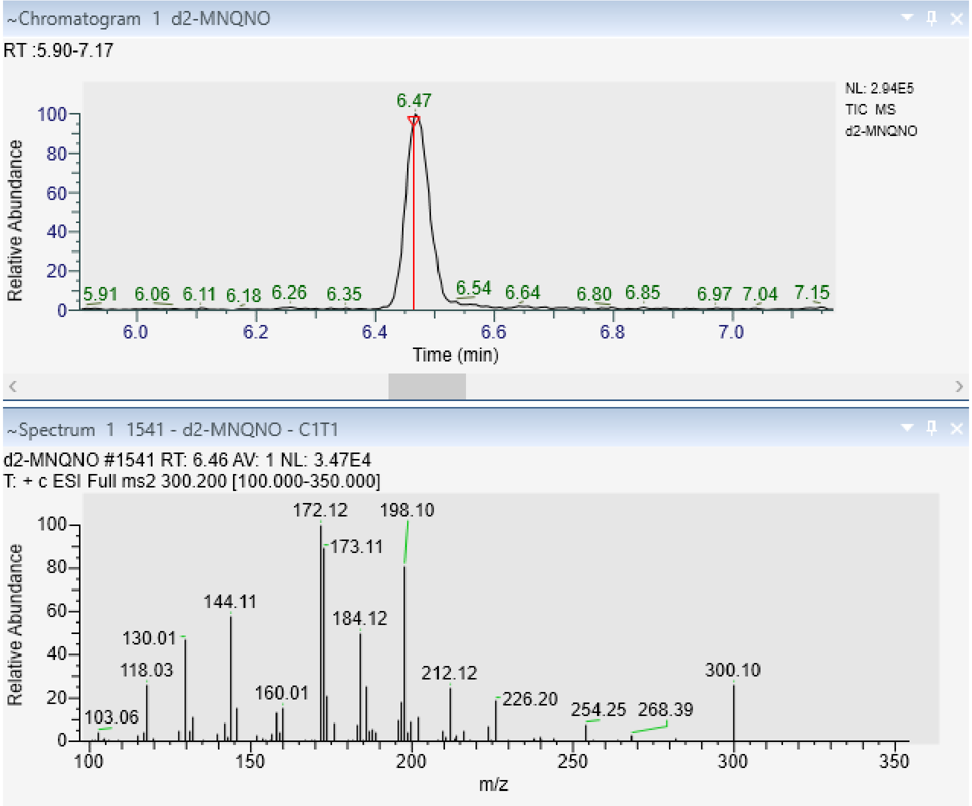

Note: For relative abundance of detected fragments see Table S3. For proposed structures of fragment ions see the next page.

Proposed structures of fragment ions

For AQs:

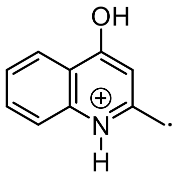

calc. m/z: 159.0679

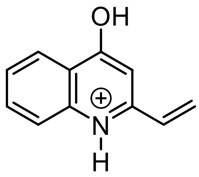

calc. m/z: 172.0757

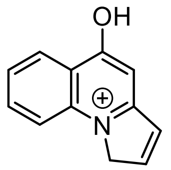

calc. m/z: 184.0757

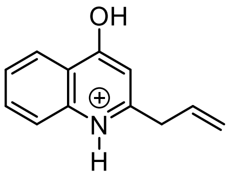

calc. m/z: 186.0914

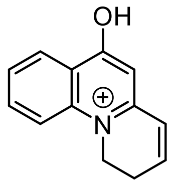

calc. m/z: 198.0914

For MAQs:

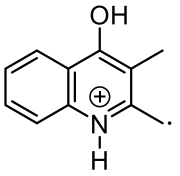

calc. m/z: 173.0836

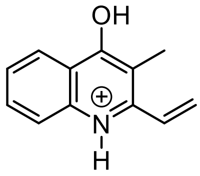

calc. m/z: 186.0914

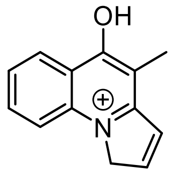

calc. m/z: 198.0914

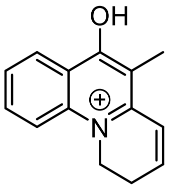

calc. m/z: 212.1070

Extracts analysis (Selected reaction monitoring (SRM))

Δ^2-NQ in ***C. tropicus*** (RT = 5.81, Mass transition 270/184)

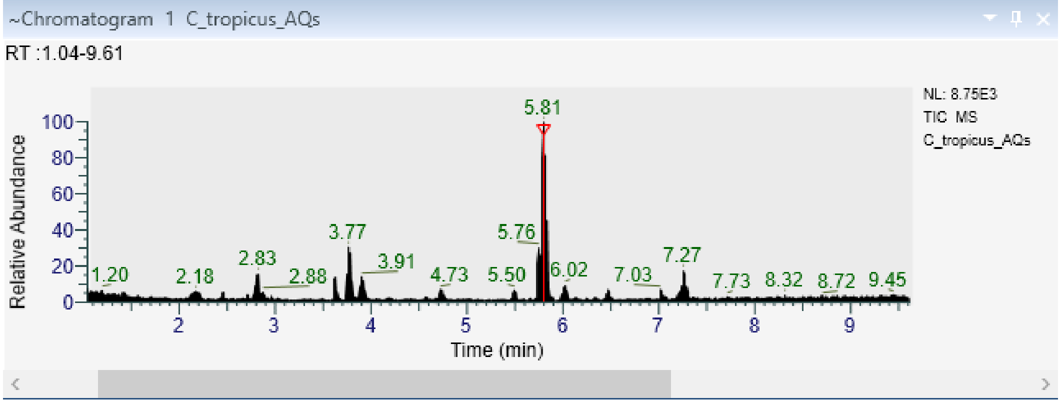

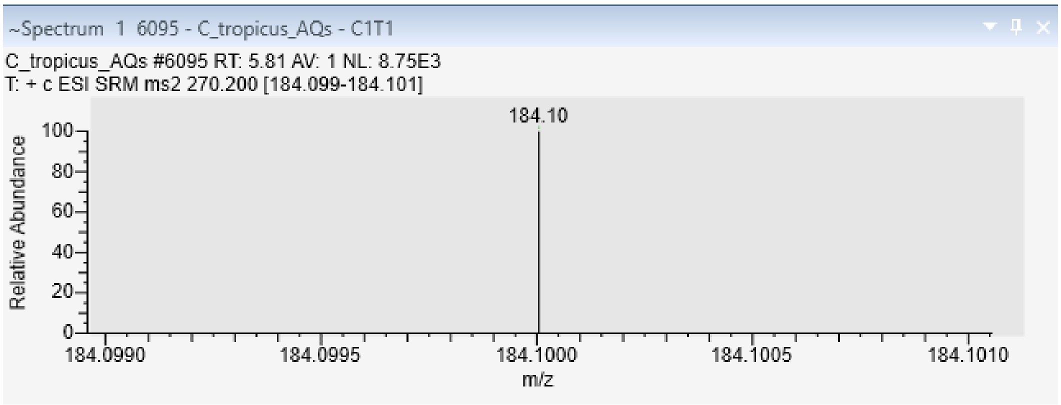

Δ^2-MNQ in ***C. tropicus*** (RT = 6.53, Mass transition 284/173)

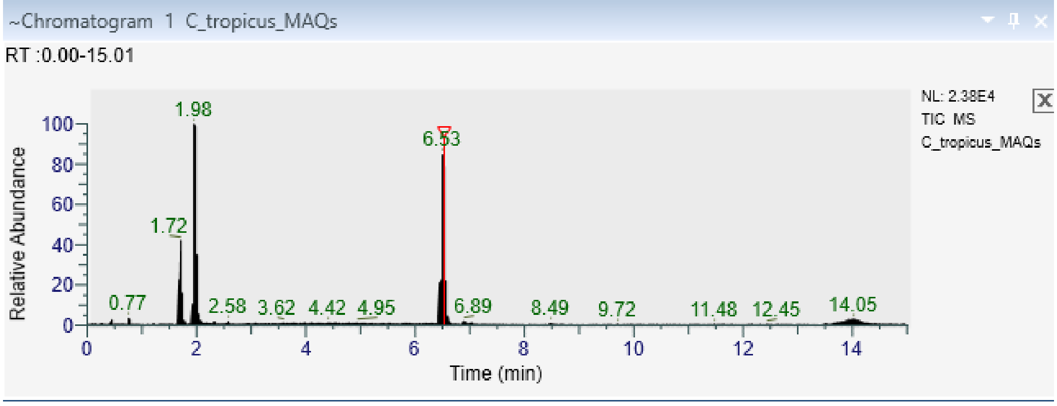

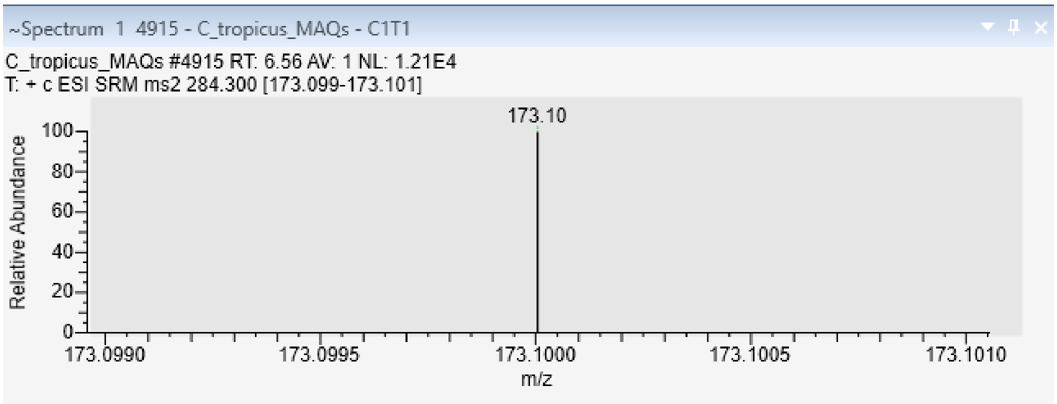

Δ^{2}-MNQNO in ***C. tropicus*** (RT = 6.48, Mass transition 300/173)

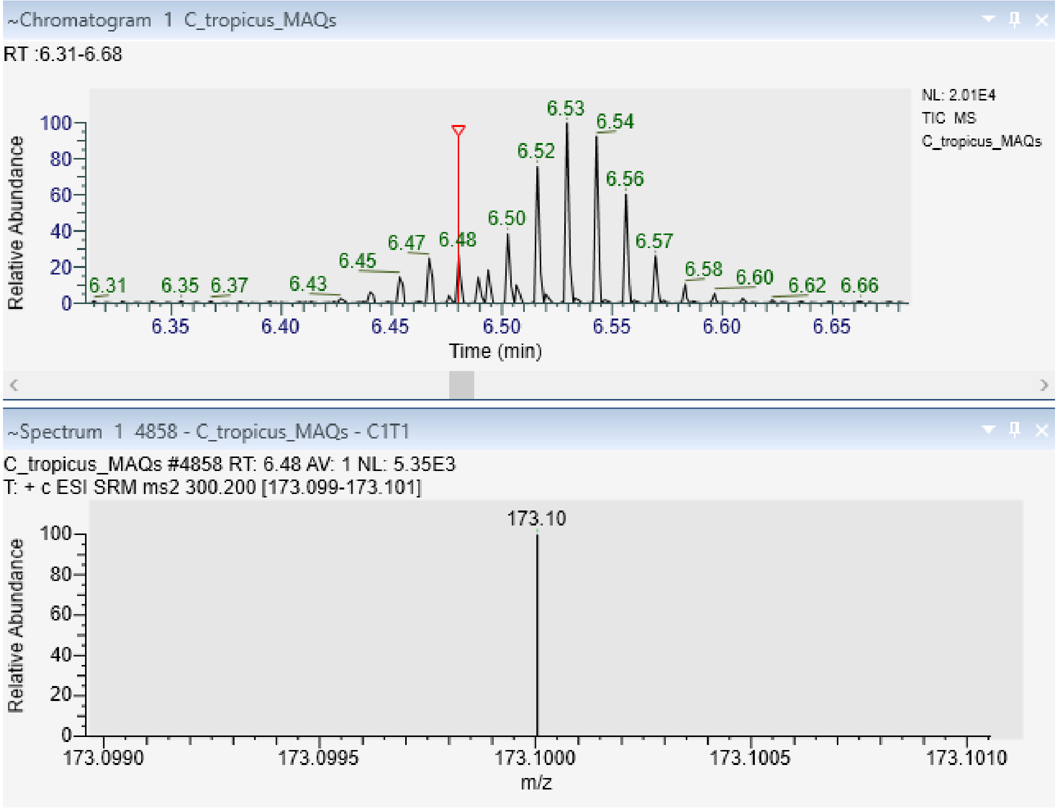

HHQ in ***B. pyrrocinia*** (RT = 4.73, Mass transition 244/159)

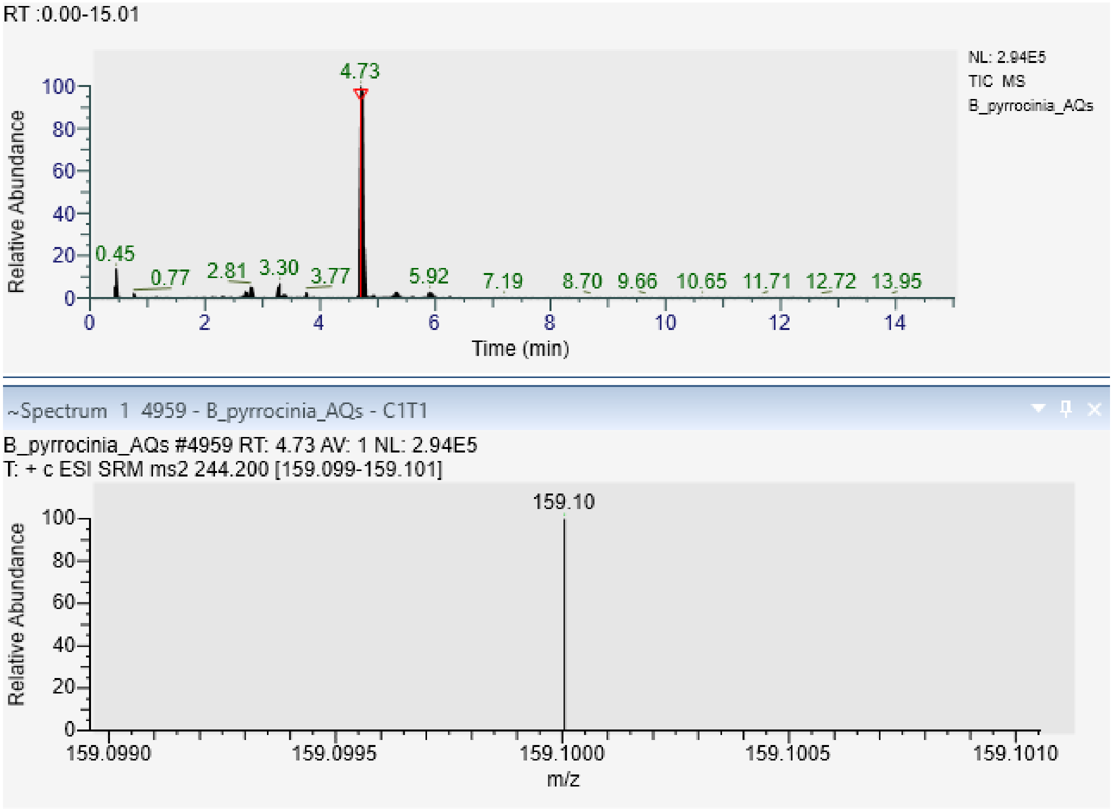

MHQ in ***B. pyrrocinia*** (RT = 5.62, Mass transition 258/173)

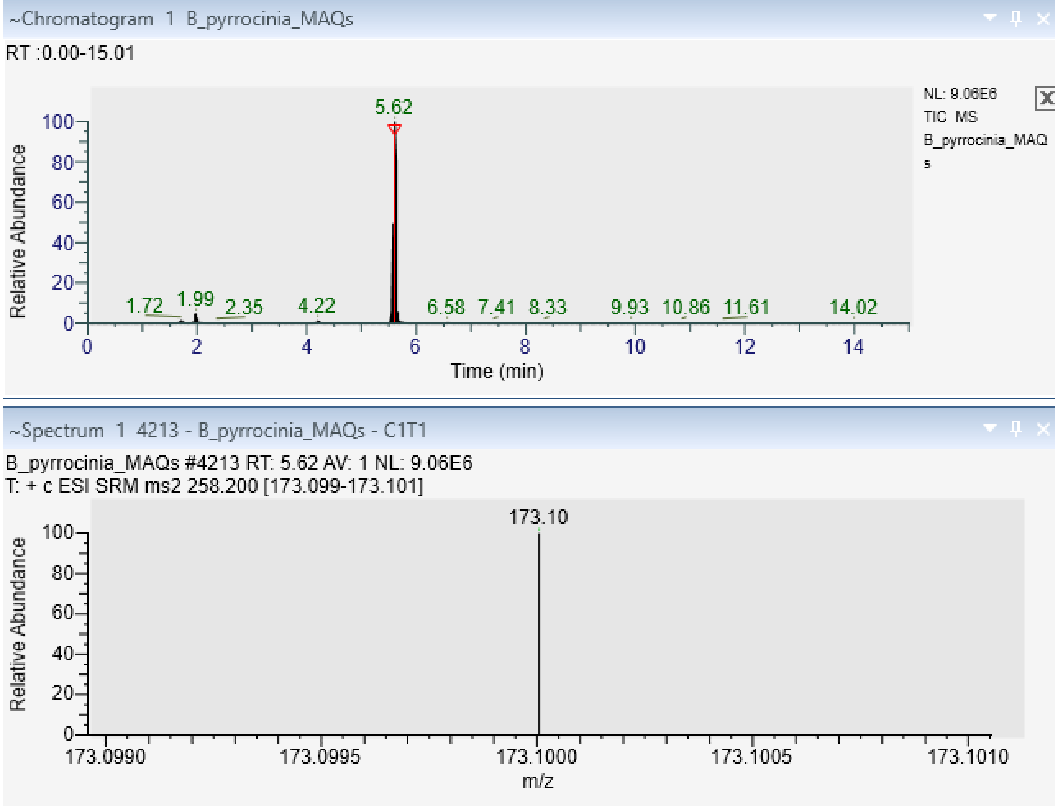

Δ^2-MNQ in ***B. pyrrocinia*** (RT = 6.58, Mass transition 284/173)

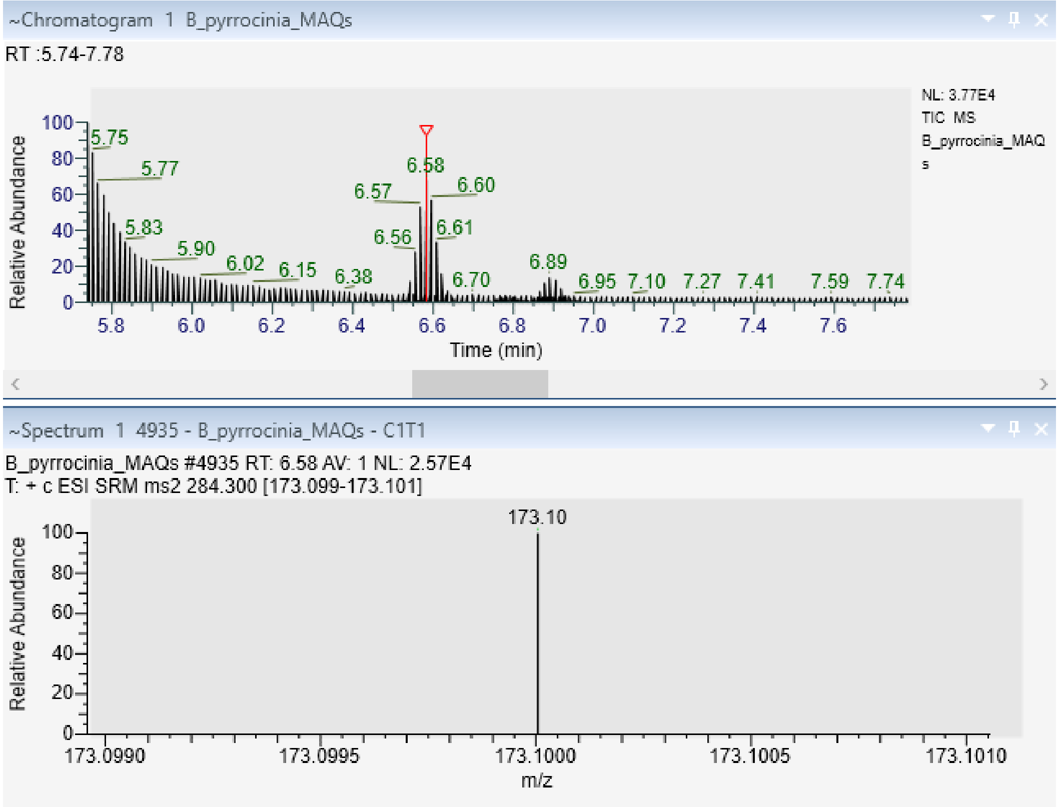

MNQ in ***B. pyrrocinia*** (RT = 6.89, Mass transition 286/173)

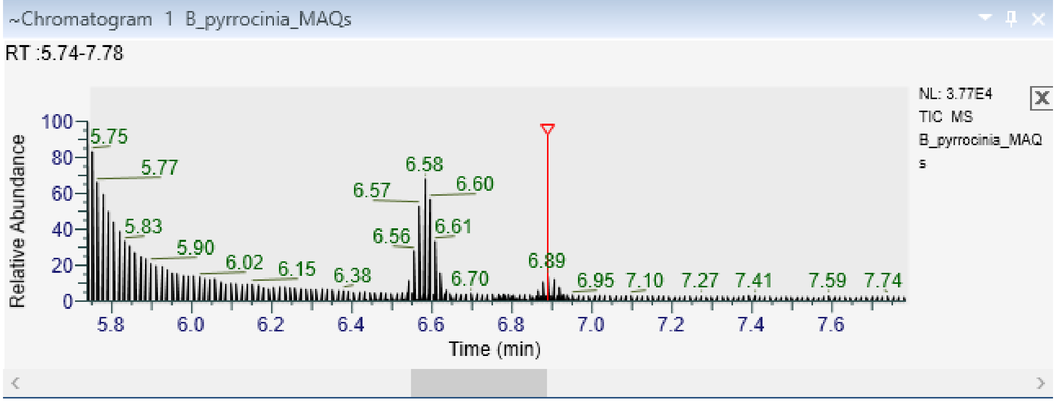

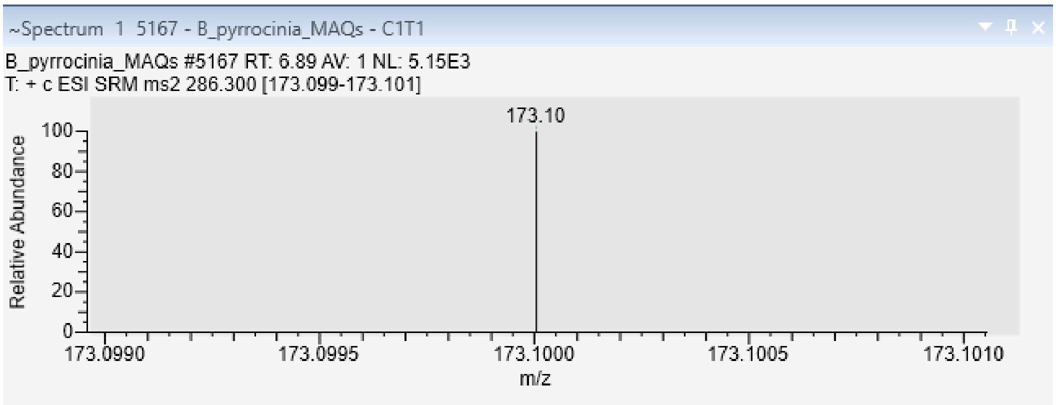

3. Tables

Table S1. Bacterial strains and their growth conditions.

Strain	Growth conditions
Chitinivorax tropicus (DSM27165)	Medium 830; 37˚C; 180 rpm
Burkholderia pyrrocinia (DSM10685)	LB medium; 37˚C; 180 rpm
Pseudomonas alcaligenes (DSM50342)	LB medium; 37˚C; 180 rpm

Table S2. Calibration curves for quantification of 4-quinolones.

Calibration standard	RT	Mass transitions	Calibration equation; R-value; Concentrations, [ng/mL]	LLOQ, [ng/mL]	LOD, [ng/mL]
2-heptyl-4-quinolone (HHQ)	4.78	244/159	Y = 544474+195647*X; R^2 = 0.9986; C = 0.032, 0.16, 0.80, 4.00, 20.00, 100.00, 500.00	0.032 (0.13 nM)	0.003
trans-Δ^2-unsaturated 2-nonyl-4-quinolone (Δ^2-NQ)	5.81	270/184	Y = 59741.2+28816.6*X; R^2 = 0.9988; C = 0.032, 0.16, 0.80, 4.00, 20.00, 100.00, 500.00	0.032 (0.12 nM)	0.003
3-methyl-2-nonyl-4-quinolone (MNQ)	6.95	286/173	Y = 38479.5+68374.2*X; R^2 = 0.9954; C = 0.032, 0.16, 0.80, 4.00, 20.00, 100.00, 500.00	0.032 (0.11 nM)	0.003
trans-Δ^2-unsaturated 3-methyl-2-nonyl-4-quinolone (Δ^2-MNQ)	6.53	284/173	Y = 116517+105164*X; R^2 = 0.9990; C = 0.032, 0.16, 0.80, 4.00, 20.00, 100.00, 500.00	0.032 (0.11 nM)	0.003
trans-Δ^2-unsaturated 3-methyl-2-nonyl-4-quinolone *N*-oxide (Δ^2-MNQNO)	6.47	300/173	Y = 1269.75+14154.1*X; R^2 = 0.9970; C = 0.032, 0.16, 0.80, 4.00, 20.00, 100.00, 500.00	0.032 (0.11 nM)	0.003

Table S3. Relative abundance of detected fragments of each standard after fragmentation (CID=30 V).

Standard	RT	Parental mass	Fragments (m/z and relative abundance)
HHQ	4.71-78	288	159 (100%), 270 (37%), 172 (35%), 184 (20%), 198 (15%)
Δ^2-NQ	5.79-5.81	270	159 (100%), 172 (25%), 184 (15%), 198 (12%)
Δ^2-MNQ	6.52-6.58	284	173 (100%), 184 (20%), 186 (17%), 198 (12%), 284 (18%)
Δ^2-MNQNO	6.47-6.48	300	173 (100%), 172 (97%), 184 (65%), 198 (88%), 212 (32%), 300 (33%)
MNQ	6.88-6.89	286	286 (100%), 173 (75%), 186 (20%)

Table S4. Query coverage and percent identity of *Pseudomonas aeruginosa* PAO1 homologs in *Pseudoalteromonas galatheae*, *Burkholderia thailandensis*, and *Chitinivorax tropicus*.

Protein of ***Pseudo-monas aeruginosa* PAO1**	Query coverage of the homolog in ***Pseudo-alteromonas galatheae***	Query coverage of the homolog in ***Burkholderia thailandensis***	Query coverage of the homolog in ***Chitinivorax tropicus***	Percent identity of the homolog in ***Pseudo-alteromonas galatheae***	Percent identity of the homolog in ***Burkholderia thai-landensis***	Percent identity of the homolog in ***Chitinivorax tropicus***
pqsA	97 %	88 %	90 %	38.51 %	30.70 %	31.21 %
pqsB	36 %	32 %	45 %	28.30 %	41.76 %	38.46 %
pqsC	99 %	99 %	98 %	29.89 %	38.59 %	36.83 %
pqsD	96 %	96 %	96 %	48.81 %	54.29 %	54.60 %
pqsE	97 %	98 %	95 %	31.58 %	31.56 %	32.08 %
phnA	70 %	-	-	36.41 %	-	-
phnB	95 %	-	-	41.71 %	-	-
pqsL	78 %	96 %	95 %	51.17 %	20.62 %	56.84 %

Table S5. Δ^2-MNQNO detection in bacterial extracts. ND = not detected.

a

**Sample**					**Integrated area**
**Bacterium**	**Incubation time, [h]**	**Incubation temperature, [°C]**	**Concentrated**	**Replicate**	
C. tropicus / B. pyrrocinia	9	37	x1	1	ND
C. tropicus / B. pyrrocinia	9	37	x1	2	ND
C. tropicus / B. pyrrocinia	13	37	x1	1	ND
C. tropicus / B. pyrrocinia	13	37	x1	2	ND
C. tropicus / B. pyrrocinia	18	37	x1	1	ND
C. tropicus / B. pyrrocinia	18	37	x1	2	ND
C. tropicus / B. pyrrocinia	21	37	x1	1	ND
C. tropicus / B. pyrrocinia	21	37	x1	2	ND
C. tropicus / B. pyrrocinia	24	37	x1	1	ND
C. tropicus / B. pyrrocinia	24	37	x1	2	ND

b

Sample					Integrated area	Average Integrated area	Concen-tration
**Bacterium**	**Incubation time, [h]**	**Incubation tempe-rature, [°C]**	**Concen-trated**	**Replicate**			
C. tropicus	22	37	x12	1	18142	22507	0.13 µg L^{-1} or 0.42 nM
C. tropicus	22	37	x12	2	26872		
B. pyrro-cinia	22	37	x12	1	ND	ND	ND
B. pyrro-cinia	22	37	x12	2	ND		
B. pyrro-cinia	22	37	x12	3	ND		

Table S6. Δ^2-MNQ detection in bacterial extracts. ND = not detected. LLOQ = Lower limit of quantification.

a

Sample					Integrated area
**Bacterium**	**Incubation time, [h]**	**Incubation temperature, [°C]**	**Concentrated**	**Replicate**	
C. tropicus / B. pyrrocinia	9	37	x1	1	ND
C. tropicus / B. pyrrocinia	9	37	x1	2	ND
C. tropicus / B. pyrrocinia	13	37	x1	1	ND
C. tropicus / B. pyrrocinia	13	37	x1	2	ND
C. tropicus / B. pyrrocinia	18	37	x1	1	ND
C. tropicus / B. pyrrocinia	18	37	x1	2	ND
C. tropicus / B. pyrrocinia	21	37	x1	1	ND
C. tropicus / B. pyrrocinia	21	37	x1	2	ND
C. tropicus / B. pyrrocinia	24	37	x1	1	ND
C. tropicus / B. pyrrocinia	24	37	x1	2	ND

b

Sample					Integrated area	Average Integrated area	Concentration
**Bacterium**	**Incubation time, [h]**	**Incubation temperature, [°C]**	**Concentrated**	**Replicate**			
C. tropicus	22	37	x12	1	68626	67375	<LLOQ
C. tropicus	22	37	x12	2	66124		
B. pyrrocinia	22	37	x12	1	145818	114499	<LLOQ
B. pyrrocinia	22	37	x12	2	88064		
B. pyrrocinia	22	37	x12	3	109615		

Table S7. MNQ detection in bacterial extracts. ND = not detected. LLOQ = Lower limit of quantification.

a

Sample					Integrated area
**Bacterium**	**Incubation time, [h]**	**Incubation temperature, [°C]**	**Concentrated**	**Replicate**	
C. tropicus / B. pyrrocinia	9	37	x1	1	ND
C. tropicus / B. pyrrocinia	9	37	x1	2	ND
C. tropicus / B. pyrrocinia	13	37	x1	1	ND
C. tropicus / B. pyrrocinia	13	37	x1	2	ND
C. tropicus / B. pyrrocinia	18	37	x1	1	ND
C. tropicus / B. pyrrocinia	18	37	x1	2	ND
C. tropicus / B. pyrrocinia	21	37	x1	1	ND
C. tropicus / B. pyrrocinia	21	37	x1	2	ND
C. tropicus / B. pyrrocinia	24	37	x1	1	ND
C. tropicus / B. pyrrocinia	24	37	x1	2	ND

b

Sample					Integrated area	Average Integrated area	Concen-tration
**Bacterium**	**Incubation time, [h]**	**Incubation tempe-rature, [°C]**	**Concen-trated**	**Replicate**			
B. pyrro-cinia	22	37	x12	1	31849	26496	<LLOQ
B. pyrro-cinia	22	37	x12	2	22186		
B. pyrro-cinia	22	37	x12	3	25454		
C. tropicus	22	37	x12	1	ND	ND	ND
C. tropicus	22	37	x12	2	ND		

Table S8. MHQ detection in bacterial extracts. ND = not detected.

a

Sample					Integrated area
**Bacterium**	**Incubation time, [h]**	**Incubation temperature, [°C]**	**Concentrated**	**Replicate**	
C. tropicus / B. pyrrocinia	9	37	x1	1	ND
C. tropicus / B. pyrrocinia	9	37	x1	2	ND
C. tropicus / B. pyrrocinia	13	37	x1	1	ND
C. tropicus / B. pyrrocinia	13	37	x1	2	ND
C. tropicus / B. pyrrocinia	18	37	x1	1	ND
C. tropicus / B. pyrrocinia	18	37	x1	2	ND
C. tropicus / B. pyrrocinia	21	37	x1	1	ND
C. tropicus / B. pyrrocinia	21	37	x1	2	ND
C. tropicus / B. pyrrocinia	24	37	x1	1	ND
C. tropicus / B. pyrrocinia	24	37	x1	2	ND

b

Sample					Integrated area	Average Integrated area	Concen-tration
**Bacterium**	**Incubation time, [h]**	**Incubation tempe-rature, [°C]**	**Concen-trated**	**Replicate**			
B. pyrro-cinia	22	37	x12	1	42986579	36535867	*44.48 µg L^{-1} or 172.82 nmol
B. pyrro-cinia	22	37	x12	2	29777529		
B. pyrro-cinia	22	37	x12	3	36843494		
C. tropicus	22	37	x12	1	ND	ND	ND
C. tropicus	22	37	x12	2	ND		

* Quantified based on the calibration curve of the closest quinolone standard (MNQ).

Table S9. Δ^2-NQ detection in bacterial extracts. ND = not detected. LLOQ = Lower limit of quantification.

a

Sample					Integrated area
**Bacterium**	**Incubation time, [h]**	**Incubation temperature, [°C]**	**Concentrated**	**Replicate**	
C. tropicus / B. pyrrocinia / P. alcaligenes	9	37	x1	1	ND
C. tropicus / B. pyrrocinia / P. alcaligenes	9	37	x1	2	ND
C. tropicus / B. pyrrocinia / P. alcaligenes	13	37	x1	1	ND
C. tropicus / B. pyrrocinia / P. alcaligenes	13	37	x1	2	ND
C. tropicus / B. pyrrocinia / P. alcaligenes	18	37	x1	1	ND
C. tropicus / B. pyrrocinia / P. alcaligenes	18	37	x1	2	ND
C. tropicus / B. pyrrocinia / P. alcaligenes	21	37	x1	1	ND
C. tropicus / B. pyrrocinia / P. alcaligenes	21	37	x1	2	ND
C. tropicus / B. pyrrocinia / P. alcaligenes	24	37	x1	1	ND

C. tropicus / B. pyrrocinia / P. alcaligenes	24	37	x1	2	ND

b

Sample					Integrated area	Average Integrated area	Concen-tration
Bacterium	Incubation time, [h]	Incubation tempe-rature, [°C]	Concen-trated	Replicate			
C. tropicus	22	37	x12	1	30640	29195	<LLOQ
C. tropicus	22	37	x12	2	27749		
B. pyrro-cinia / P. alcaligenes	22	37	x12	1	ND	ND	ND
B. pyrro-cinia / P. alcaligenes	22	37	x12	2	ND		
B. pyrro-cinia / P. alcaligenes	22	37	x12	3	ND		

Table S10. HHQ detection in bacterial extracts. ND = not detected.

a

Sample					Integrated area
Bacterium	Incubation time, [h]	Incubation temperature, [°C]	Concentrated	Replicate	
C. tropicus / B. pyrrocinia / P. alcaligenes	9	37	x1	1	ND
C. tropicus / B. pyrrocinia / P. alcaligenes	9	37	x1	2	ND
C. tropicus / B. pyrrocinia / P. alcaligenes	13	37	x1	1	ND
C. tropicus / B. pyrrocinia / P. alcaligenes	13	37	x1	2	ND
C. tropicus / B. pyrrocinia / P. alcaligenes	18	37	x1	1	ND
C. tropicus / B. pyrrocinia / P. alcaligenes	18	37	x1	2	ND

C. tropicus / B. pyrrocinia / P. alcaligenes	21	37	x1	1	ND
C. tropicus / B. pyrrocinia / P. alcaligenes	21	37	x1	2	ND
C. tropicus / B. pyrrocinia / P. alcaligenes	24	37	x1	1	ND
C. tropicus / B. pyrrocinia / P. alcaligenes	24	37	x1	2	ND

b

Sample					Integrated area	Average Integrated area	Concen-tration
**Bacterium**	**Incubation time, [h]**	**Incubation tempe-rature, [°C]**	**Concen-trated**	**Replicate**			
B. pyrro-cinia	22	37	x12	1	1181949	1056602	0.22 µg L^{-1} or 0.91 nM
B. pyrro-cinia	22	37	x12	2	889125		
B. pyrro-cinia	22	37	x12	3	1098732		
C. tropicus	22	37	x12	1	ND	ND	ND
C. tropicus	22	37	x12	2	ND		
P. alcali-genes	22	37	x12	1	ND	ND	ND
P. alcali-genes	22	37	x12	2	ND		
P. alcali-genes	22	37	x12	3	ND		

4. Figures

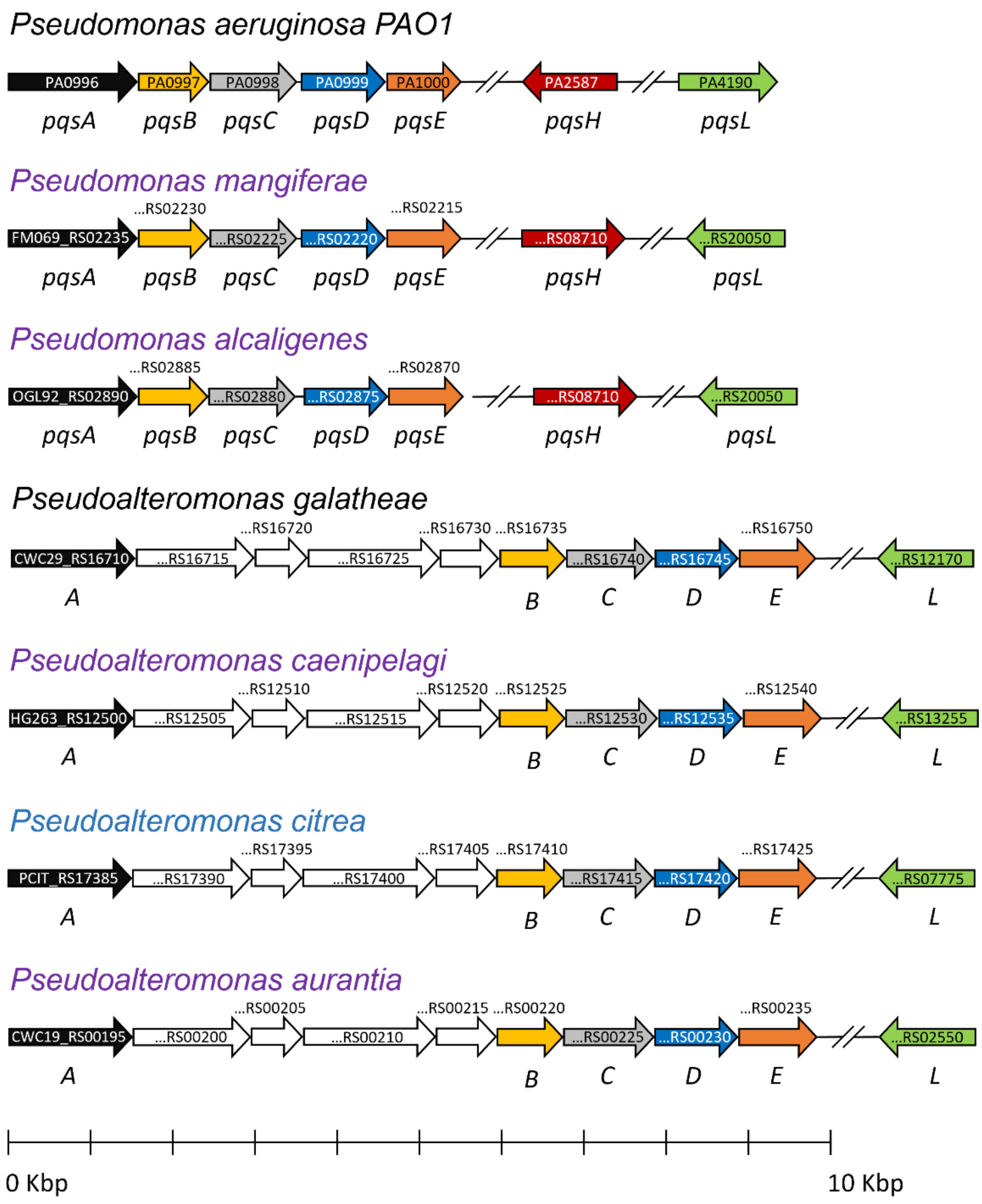

Figure S1a. Biosynthetic gene cluster (BGC) responsible for 2-alkyl-4-quinolone production in *Pseudomonas* and *Pseudoalteromonas* spp.: reported to contain BGCs and produce quinolones (species labelled black), reported to contain BGCs but were not studied for quinolone production (species labelled blue), and not reported to contain BGCs and not studied for quinolone production (species labelled violet).

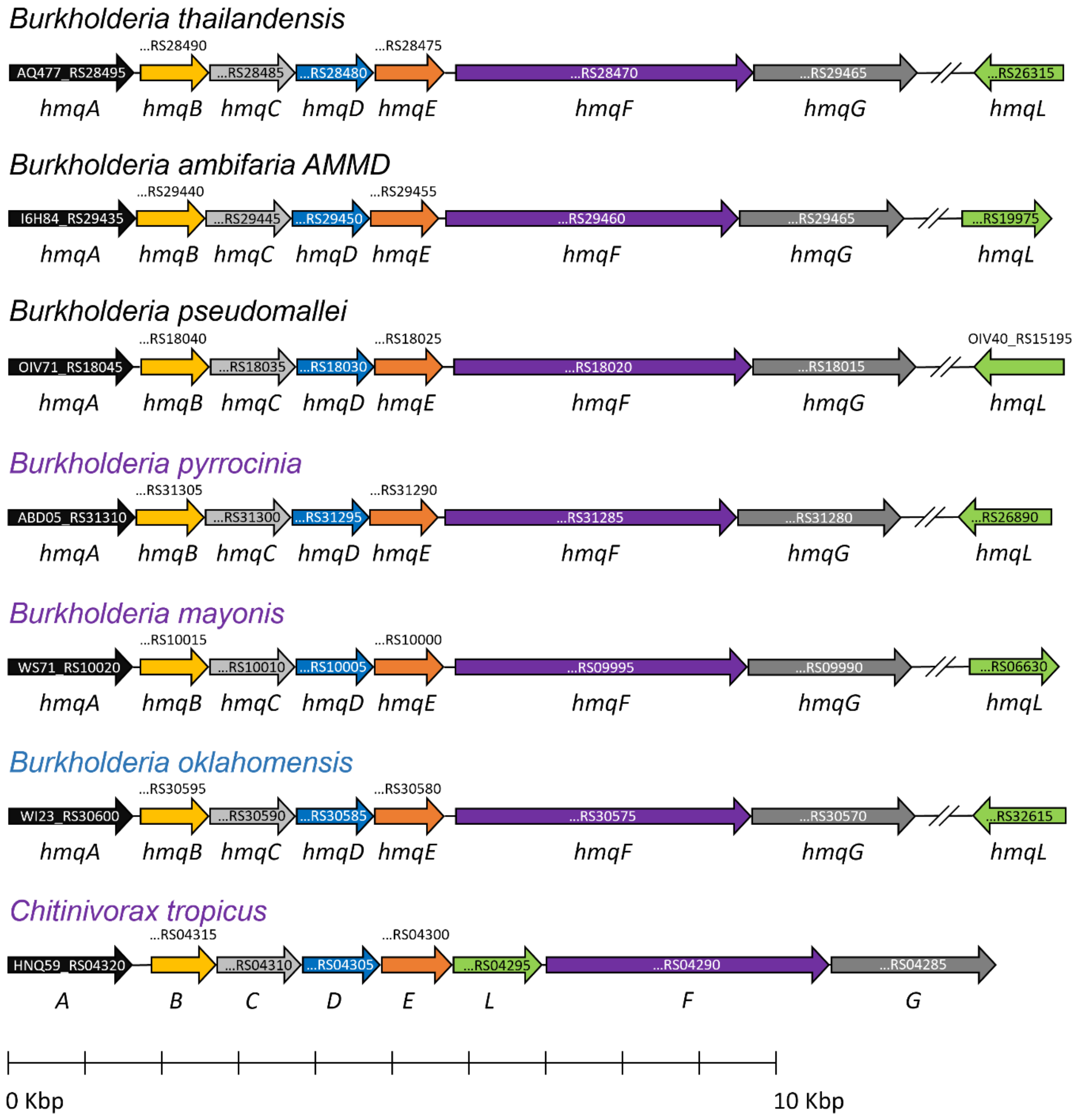

Figure S1b. Biosynthetic gene cluster (BGC) responsible for 2-alkyl-4-quinolone production in *Burkholderia* spp. and *Chitinivorax tropicus*: reported to contain BGCs and produce quinolones (species labelled black), reported to contain BGCs but were not studied for quinolone production (species labelled blue), and not reported to contain BGCs and not studied for quinolone production (species labelled violet).

Figure S2

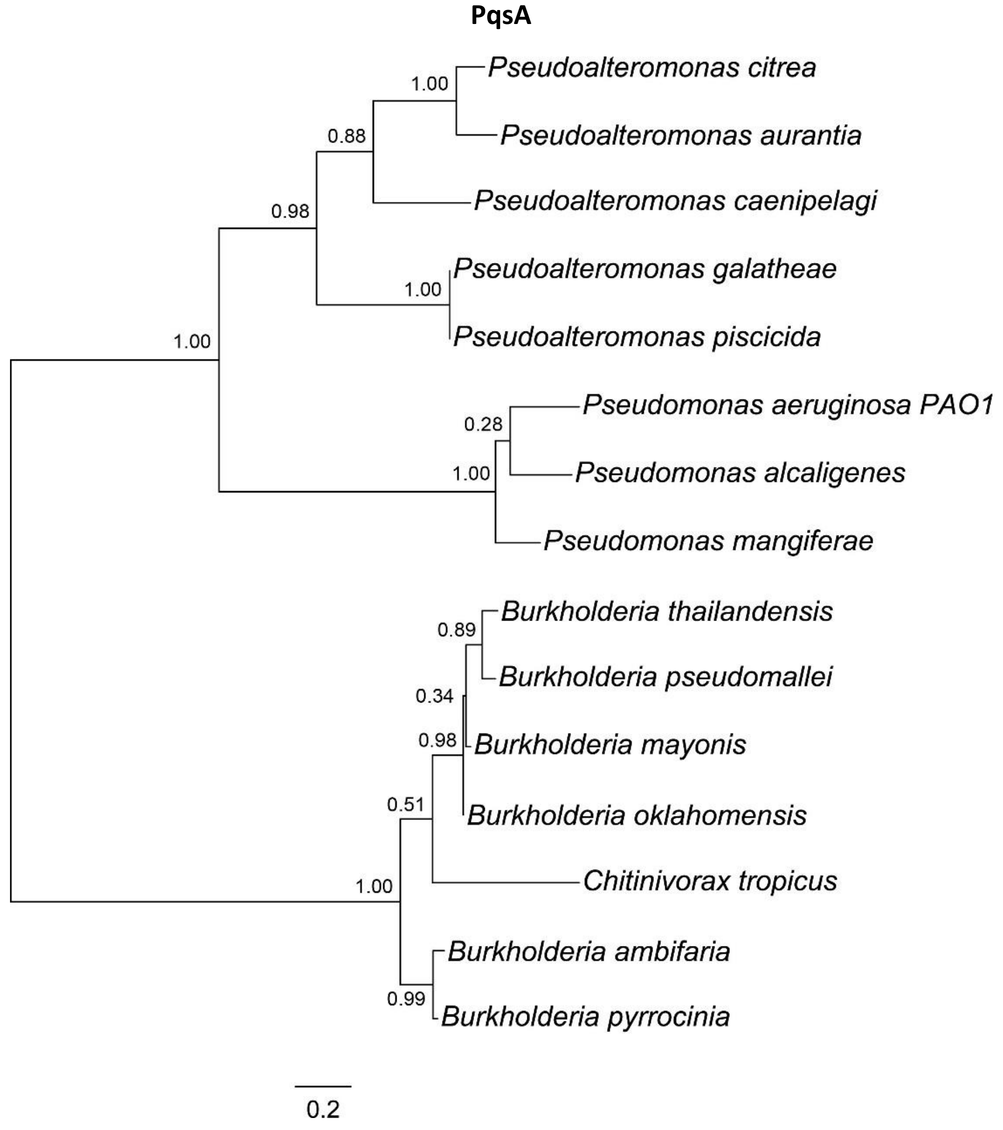
PqsA
Pseudoalteromonas citrea
1.00
Pseudoalteromonas aurantia
0.88
Pseudoalteromonas caenipelagi
0.98
Pseudoalteromonas galatheae
1.00
Pseudoalteromonas piscicida
1.00
Pseudomonas aeruginosa PAO1
0.28
Pseudomonas alcaligenes
1.00
Pseudomonas mangiferae
Burkholderia thailandensis
0.89
Burkholderia pseudomallei
0.34
0.98
Burkholderia mayonis
0.51
Burkholderia oklahomensis
Chitinivorax tropicus
1.00
Burkholderia ambifaria
0.99
Burkholderia pyrrocinia
0.2

PqsB

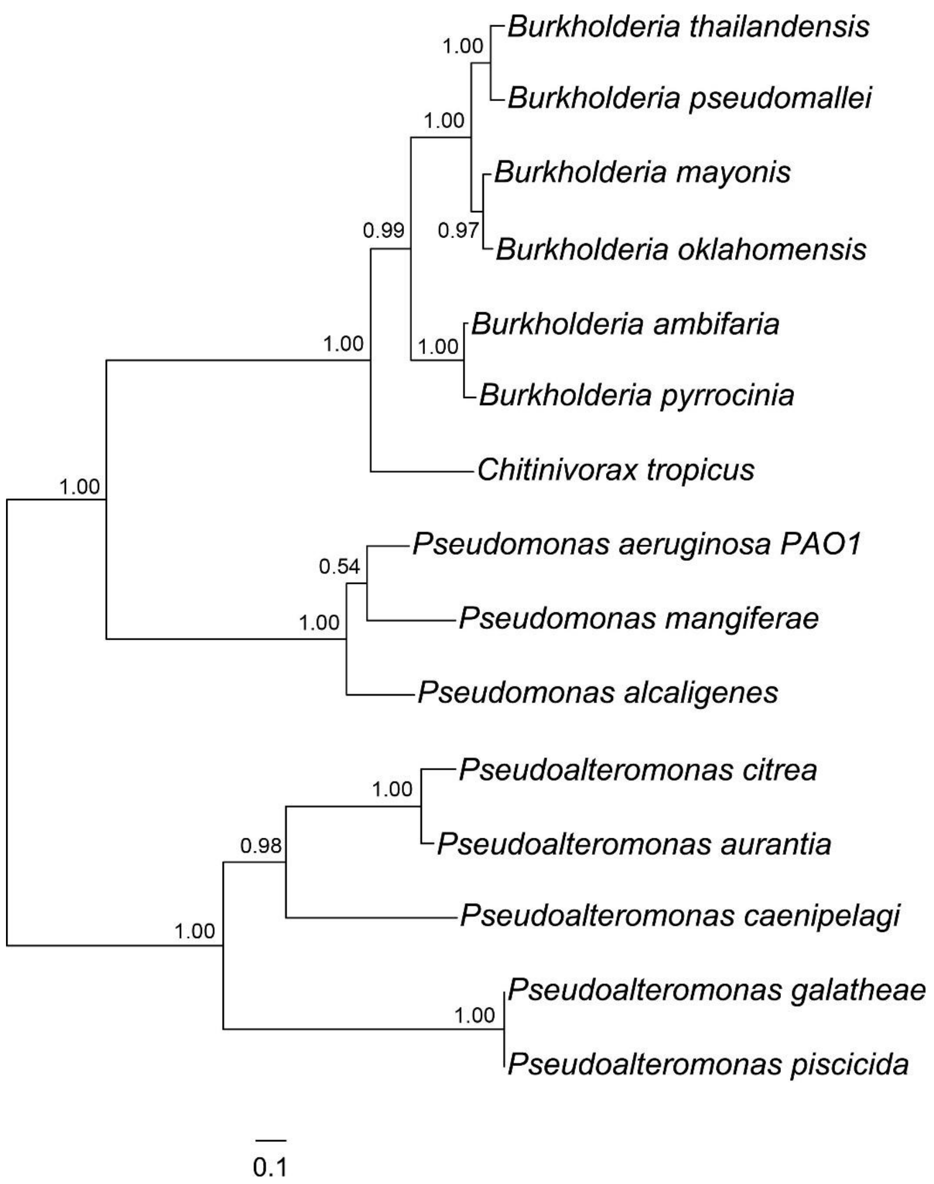
Burkholderia thailandensis
Burkholderia pseudomallei
Burkholderia mayonis
Burkholderia oklahomensis
Burkholderia ambifaria
Burkholderia pyrrocinia
Chitinivorax tropicus
Pseudomonas aeruginosa PAO1
Pseudomonas mangiferae
Pseudomonas alcaligenes
Pseudoalteromonas citrea
Pseudoalteromonas aurantia
Pseudoalteromonas caenipelagi
Pseudoalteromonas galatheae
Pseudoalteromonas piscicida
1.00
1.00
0.99
0.97
1.00
1.00
1.00
0.54
1.00
1.00
0.98
1.00
1.00
0.1

PqsC

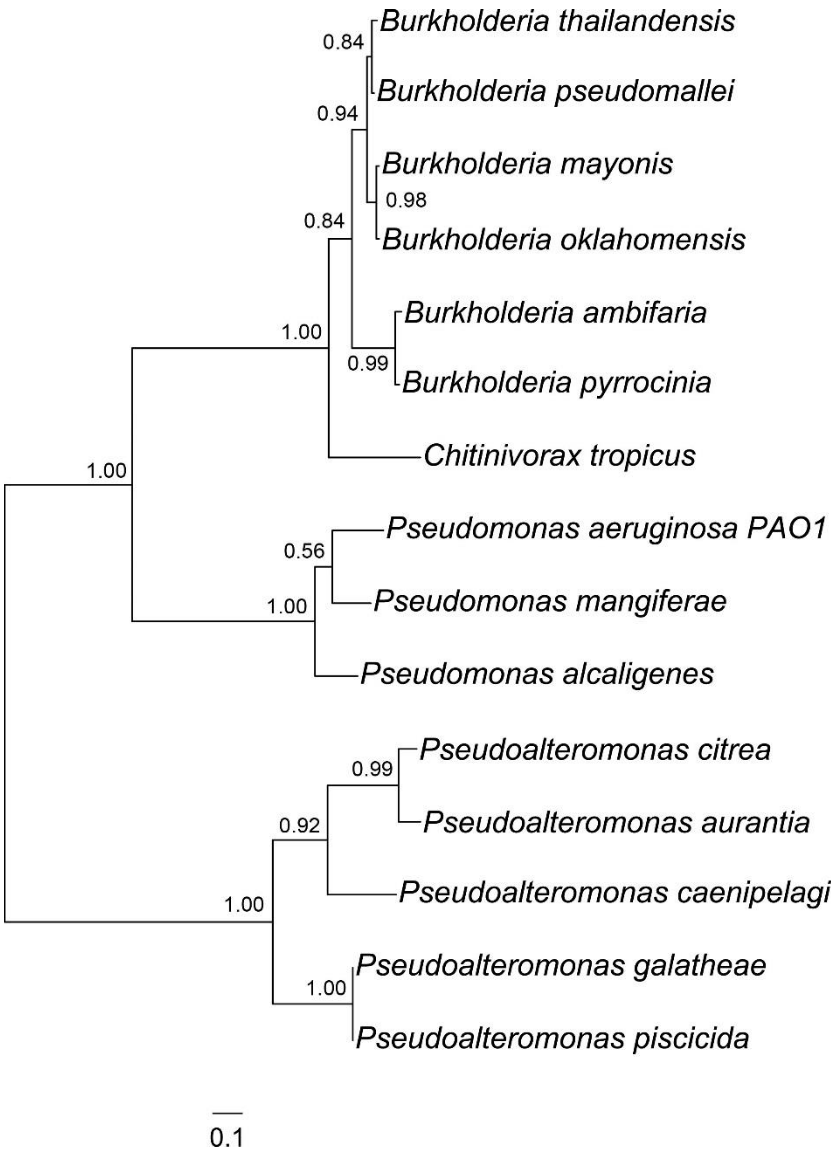
Burkholderia thailandensis
0.84
Burkholderia pseudomallei
0.94
Burkholderia mayonis
0.98
Burkholderia oklahomensis
0.84
Burkholderia ambifaria
1.00
0.99
Burkholderia pyrrocinia
Chitinivorax tropicus
1.00
Pseudomonas aeruginosa PAO1
0.56
Pseudomonas mangiferae
1.00
Pseudomonas alcaligenes
Pseudoalteromonas citrea
0.99
Pseudoalteromonas aurantia
0.92
Pseudoalteromonas caenipelagi
1.00
Pseudoalteromonas galatheae
1.00
Pseudoalteromonas piscicida
0.1

PqsD

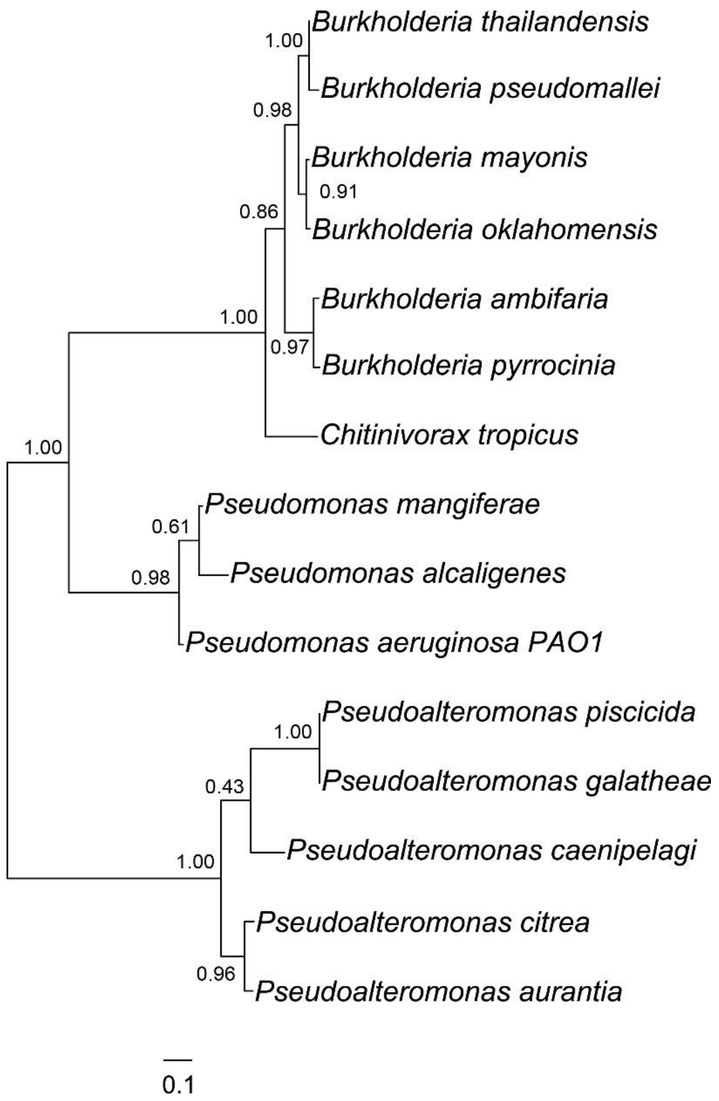

Burkholderia thailandensis
1.00
Burkholderia pseudomallei
0.98
Burkholderia mayonis
0.91
0.86
Burkholderia oklahomensis
Burkholderia ambifaria
1.00
0.97
Burkholderia pyrrocinia
Chitinivorax tropicus
1.00
Pseudomonas mangiferae
0.61
Pseudomonas alcaligenes
0.98
Pseudomonas aeruginosa PAO1
Pseudoalteromonas piscicida
1.00
0.43
Pseudoalteromonas galatheae
Pseudoalteromonas caenipelagi
1.00
Pseudoalteromonas citrea
0.96
Pseudoalteromonas aurantia
0.1

PqsE

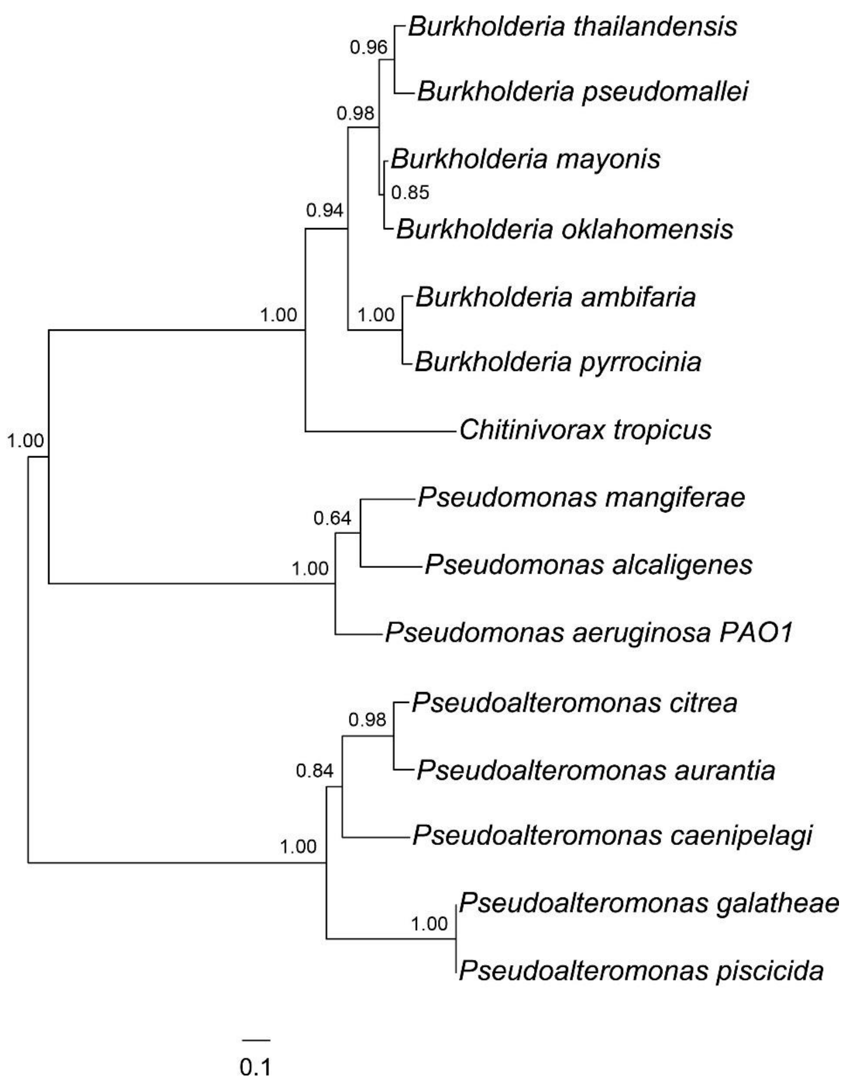

Figure S2. Phylogenetic trees based on protein sequences of PqsA, PqsB, PqsC, PqsD, or PqsE and their homologs. The evolutionary history was inferred using the maximum likelihood (ML) method and a JTT matrix-based model.[3] Bootstrap values, indicated at the nodes, were obtained from 1000 bootstrap replicates. Branches corresponding to partitions reproduced in less than 50% bootstrap replicates are collapsed. The evolutionary distances are in the units of the number of amino acid substitutions per site. All trees in the figure are midpoint rooted.

Figure S3

HmqF

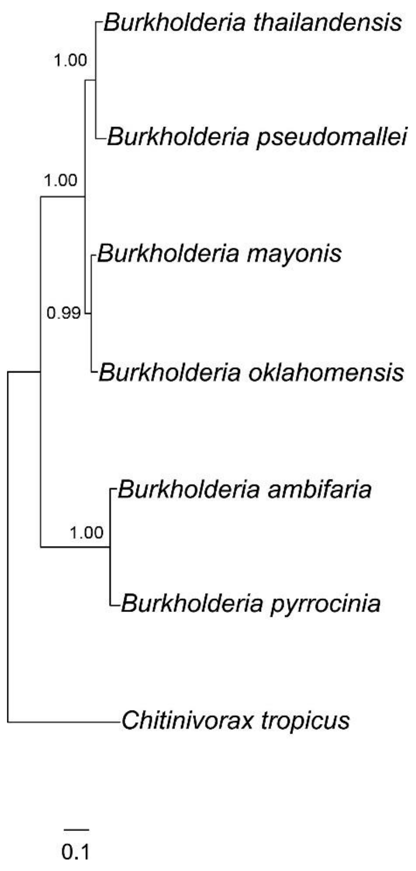

HmqG

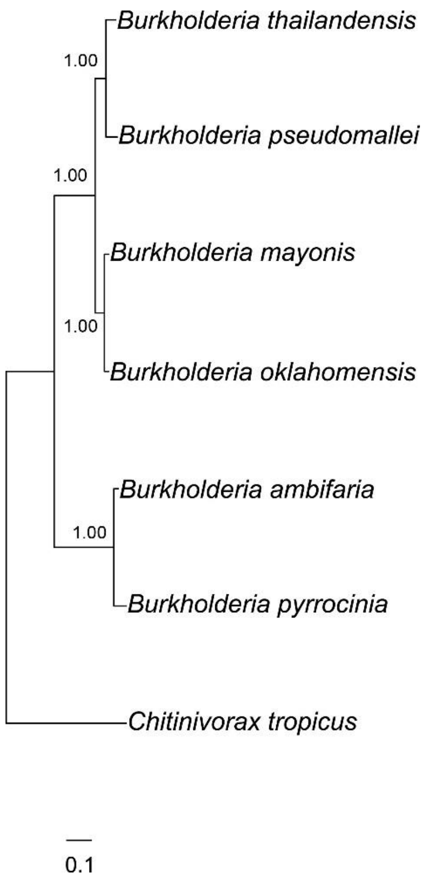

Figure S3. Phylogenetic trees based on protein sequences of HmqF or HmqG and their homologs. The evolutionary history was inferred using the maximum likelihood (ML) method and a JTT matrix-based model.[3] Bootstrap values, indicated at the nodes, were obtained from 1000 bootstrap replicates. Branches corresponding to partitions reproduced in less than 50% bootstrap replicates are collapsed. The evolutionary distances are in the units of the number of amino acid substitutions per site. Both trees in the figure are midpoint rooted.

PqsL/HmqL

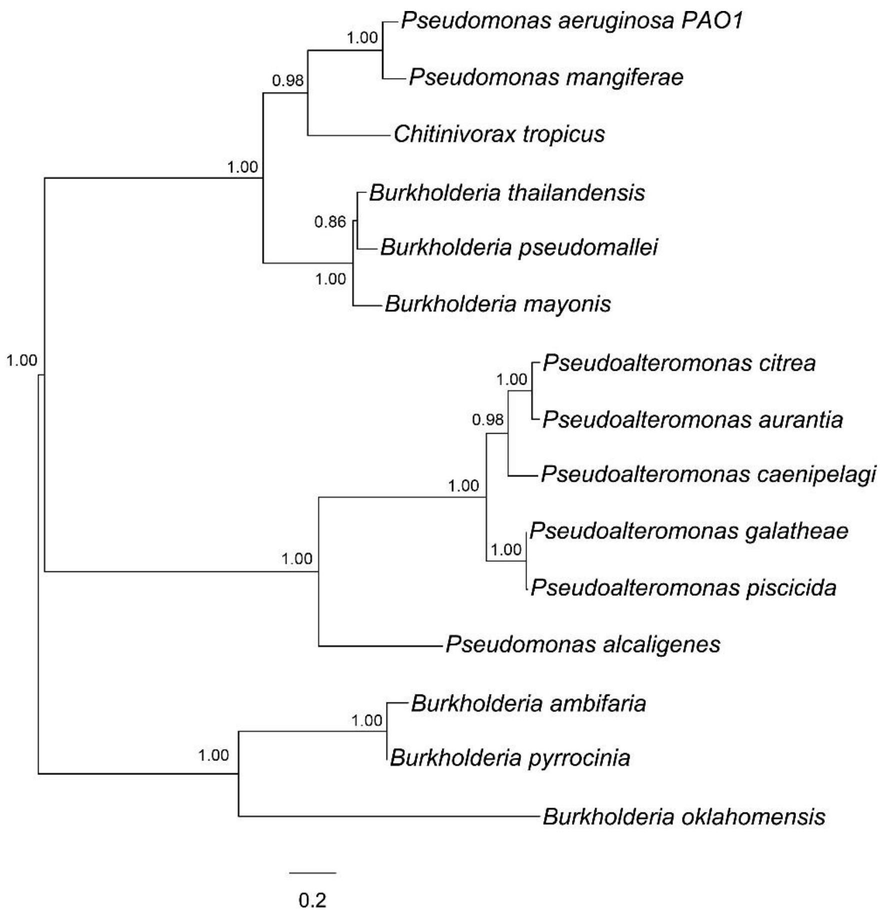

Figure S4. Midpoint rooted phylogenetic tree based on protein sequences of PqsL/HmqL homologs. The evolutionary history was inferred using the maximum likelihood (ML) method and a JTT matrix-based model.[3] Bootstrap values, indicated at the nodes, were obtained from 1000 bootstrap replicates. Branches corresponding to partitions reproduced in less than 50% bootstrap replicates are collapsed. The evolutionary distances are in the units of the number of amino acid substitutions per site.

5. References

(1) Prothiwa, M., Filz, V., Oehler, S., and Böttcher, T. (2021) Inhibiting quinolone biosynthesis of Burkholderia, *Chem Sci 12*, 6908-6912.

(2) Szamosvári, D., Prothiwa, M., Dieterich, C. L., and Böttcher, T. (2020) Profiling structural diversity and activity of 2-alkyl-4(1H)-quinolone N-oxides of Pseudomonas and Burkholderia, *Chem Commun 56*, 6328-6331.

(3) Jones, D. T., Taylor, W. R., and Thornton, J. M. (1992) The rapid generation of mutation data matrices from protein sequences, *Comput Appl Biosci 8*, 275-282.

(4) Guindon, S., Dufayard, J. F., Lefort, V., Anisimova, M., Hordijk, W., and Gascuel, O. (2010) New algorithms and methods to estimate maximum-likelihood phylogenies: assessing the performance of PhyML 3.0, *Syst Biol 59*, 307-321.

(5) Anisimova, M., and Gascuel, O. (2006) Approximate likelihood-ratio test for branches: A fast, accurate, and powerful alternative, *Syst Biol 55*, 539-552.

(6) Tamura, K., Stecher, G., and Kumar, S. (2021) MEGA11: Molecular Evolutionary Genetics Analysis Version 11, *Mol Biol Evol 38*, 3022-3027.

(7) Felsenstein, J. (1985) Confidence Limits on Phylogenies: An Approach Using the Bootstrap, *Evolution 39*, 783-791.
